# The CAMUS Initiative: A Multiphase, Multicentre International Collaboration to Redefine Risk Stratification, Reporting, and Grading of Surgical Complications in Urology

**DOI:** 10.1016/j.euros.2026.01.012

**Published:** 2026-04-06

**Authors:** Christopher Soliman, Niranjan J. Sathianathen, Niall M. Corcoran, Nathan Lawrentschuk, Patrick Y. Wuethrich, Marc A. Furrer, on behalf of the CAMUS Study Group Collaborators, Furat Abd Ali, Muhammad Fairuz Shah Bin Abd Karim, Mohamed Abouelenein, Yasmin Abu Ghanem, Ahmed Adam, Alessandro Adami, Konstantinos Adamos, Constantinos Adamou, Ademola Adeyeye, Florian Aellen, Luca Afferi, Abhishek Agarwal, Dinesh K Agarwal, Vandana Agarwal, Edoardo Agostini, Mayank Agrawal, María Ángela Agüera Sánchez, Sarfraz Ahmad, Rawdy Talaat Ahour, Ilgar Akbarov, Samira Akbas, Nebil Akdogan, Eren Erdi Aksaray, Osama Al-Bermani, Rouvier Al-Monajjed, Dheaa Al-Rammahi, David M. Albala, Peter Albers, Peter C. Albertsen, Salah Albuheissi, Hassan Aina Ali, Mohamed Ali Ibrahim Ali Mohsen, Ahmad Almarzouq, Turki Altaylouni, Reshma Ambulkar, Bastian Amend, Filip Ameye, Daniele Amparore, Yuliya Anastasiyeuskaya, Umberto Anceschi, Elliot Anderson, Paul D. Anderson, Chawat Angsurak, Jonathan Aning, Luca Antonelli, Fawad Arif, Yenny Arroyo Rojas, Luigi Ascalone, Vincenzo Asero, MHammad Ather, Jay Atkinson, Stefan Aufderklamm, Jana Luisa Aulenkamp, Riccardo Autorino, Atiqullah Aziz, Emad Aziz, Marek Babjuk, Christa Babst, Kaspar Bachmann, Stefano Cogo Badan, Ketan K. Badani, Sheela Badiger, Loïc Baekelandt, Henning Bahlburg, Craig Richard Bailey, Francesco Barletta, Mitchell Barns, Ahmad Bashir, Deepak Batura, David Beckmann, Saajida Begum, Michael Beh, Christian M Beilstein, Themistoklis Ch Bellos, Daniel Benamran, Barbora Bendova, Mattia Benedetti, Florian Beraud, Sebastian Berg, Lisa Bergauer, Frank P. Berger, Yoel Berger, Rui Miguel Marques Bernardino, Camille Berquin, Anne-Claire Berrens, Lorenzo Berti, Riccardo Bertolo, Patrick Betschart, Axel Bex, Burkhard Beyer, Jörg Beyer, Shreyas Bhadranavar, Naeem Bhojani, Alberto Bianchi, Roberto Bianchi, Uwe Bieri, Waleed Bin Ghaffar, Frédéric D. Birkhäuser, Conrad Bishop, Stefano Biundo, Syed Raziuddin Biyabani, Peter Black, James Peter Blackmur, Benjamin Blaise, Leandro Blas, Gideon Adam Blecher, Joost M. Blok, Stephan Blumenthal, Matthias Bock, Katharina Boehm, Christoph Boesing, Mehmet Salih Boğa, Eugenio Bologna, Damien Bolton, Matteo Boltri, Jochen Bongardt, Joost L. Boormans, Baba Borg, Angelika Borkowetz, Juan Boronat, Kim Borrill, Andreas Bosshard, Piet Bosshard, Silvan Boxler, Pietro Brambillasca, Anselm Bräuer, Maurizio Brausi, Carlo Andrea Bravi, Kelly K. Bree, Thomas Stefan Bregy, Alberto Briganti, Logan Briggs, Isabel Brinkmann, Mark Broe, Suzanne Broens, Stephan Brönimann, Oscar R. Brouwer, Rosannis Brown, Cliodhna Browne, Thomas Brunner, Franck Bruyere, Nicolas M Bubendorfer, Kant Buengsuchonsoontorn, Maximilian Burger, Orlando Burkhardt, Javed Burki, Kevin Gerard Byrnes, Adriana Elizabeth Cabrera Chamba, Giovanni Cacciamani, Loris Cacciatore, Declan Cahill, Gian Cajoeri, Gustavo Marcelo Caleffi Ariete, Gokhan Calik, Edward Calleja, Paolo Calzavacca, Riccardo Campi, Davide Campobasso, Geraldine Cancel-Tassin, Abdullah Erdem Canda, Luigi Candela, David Canny, Christoph Cantieni, Rosa Roberta Caporusso, Gustavo Franco Carvalhal, Alessandra Cassani, Niek F. Casteleijn, Daniele Castellani, Rafael Castilho Borges, Juan P. Cata, Rita Cataldo, Paul Cathcart, Xavier Cathelineau, James Catto, Alberto Caviglia, Valentina Ceccarelli, Valerio Cellini, Elisabetta Cerutti, Seçil Çetin, Praveen Chahar, Chu Ann Chai, Palesa Motshabi Chakane, Venu Chalasani, Benjamin Challacombe, Ramandeep Chalokia, Christos Chamos, Albert Kam Ming Chan, Kimberely Chan, Kittipong Chaowarat, Kapil Chaudhary, Lucas Arrais Chaves Nascimento, Yue Che, Joseph G Cheaib, Enrico Checcucci, Karen Chen, Kenneth Chen, Pinxia Chen, Yu-Chen Chen, But Sing Maxime Cheng, Giuseppe Chiacchio, Ijeoma Chibuzo, Sarah Chieveley-Williams, Mallikarjuna Chiruvella, Ken Chow, George Christodoulides, Paul H. Chung, Andrea Cicconofri, Antonio Cicione, Luca Cioccari, María Elena Civeira Marín, Francesco Claps, David Clarke, Silvia Clauser, Miquel Coca-Martinez, Jasamine Coles-Black, William Collier, Evi Comploj, Samantha Conroy, Shane Considine, Roberto Contieri, Niall Corcoran, Andrea Cortegiani, Stefano Corti, Elisabetta Costantini, Geoff Coughlin, Richard Coulthard, Marco Covotta, Alessandro Crestani, Vito Giuseppe Crimi, Marcus G Cumberbatch, Olivier Cussenot, David D’Andrea, Simone D’Annunzio, Carolina D’Elia, Giulia D’Ippolito, Anahita Dabo-Trubelja, Britt-Inger Dahlin, Torsten Kurt Dammann, Danny Darlington, Christopher Darr, Prokar Dasgupta, I. Datu, Valentina Daviddi, Maria Vittoria De Angelis, Ruben De Groote, Jean de la Rosette, Geert De Naeyer, Nikolaas De Neve, Daniele De Nicolo, Cosimo De Nunzio, Hielke Martijn de Vries, Noemi Deanesi, Karel Decaestecker, Stephan Degener, Peter Dekuyper, Rocco Francesco Delle Fave, Paolo Dell’Oglio, Devang Desai, Mihir Desai, Rohit R. Deshbhratar, Brecht Devos, Andrew Deytrikh, Ankita Dhir Bhasin, Bert Dhondt, Stefano Di Bari, Ida Di Giacinto, Fabrizio Di Maida, Giulia Di Marco, Mariana Dias Capinha, Jorge Díaz-Méndez, Andrew Dickinson, Dovile Diktanaite, Colin P.N. Dinney, Jeson R. Doctor, Lachlan Dodds, Anna Dodi, Ella Doerge, Scott Donnellan, Ned Douglas, Desiree Louise Draeger, Nici Markus Dreger, Giovanni Drocchi, Michael J. Droller, Danelo Estienne du Plessis, Chantal Ducret, Lorenzo Dugato, Philip Dundee, Richard Dutton, Benjamin Eddy, Christopher Eden, Luisa Egen, Martin Lukas Egger, Sarah Einerhand, Berk Yasin Ekenci, Ahmed Mohamed Aly El-Assmy, Kariem El-Boghdadly, Mostafa Nasr Mohamed Elgamal, Ahmed Elsherbiny Elkarta, Sandy Elmer, Ramzy Elnabarawy, Ines Elsemann, Samar Elshahat Abd Elaziz Saleh, Dean Elterman, Dominique A. Engel, Daniel Stephan Engeler, Simon Udo Engelmann, Thomas Enzmann, Ismail Eralp, Francesco Esperto, Irene Esposito, Tara Etherington, Wouter Everaerts, Osama Ezzat, Faith N.F. Factora, Fabian Falkenbach, Andrea Falvo, Christian Daniel Fankhauser, Pouriya Faraj Tabrizi, Gabriel Faria-Costa, Christian Thomas Favoccia, Marissa Ferguson, Stefania Ferretti, Vincenzo Ficarra, Stefanie Fiechter, Deborah Fimognari, Damien Finniss, Neil Fleshner, Decio Maria Folchini, Thomas Roshan Fonseka, Patrice Forget, Yves Fradet, Antonio Franco, Martin Franko, Henrik Fredrich, Pedro FS Freitas, Andreas Frey, Mark Frydenberg, Vittorio Fulcoli, Marc Alain Furrer, Andrew Gabrielson, Pier Paolo Gaglioti, Antonio Galfano, Sebastian Gallina, Giacomo Gallo, Sophia Gallo, Venkata Ganesh, Gauri Raman Gangakhedkar, Herney Andres Garcia-Perdomo, Giulia Garelli, Rakesh Garg, Moritz Gatscher, Vineet Gauhar, Aikaterini Gavra-Papaspyrou, Gaetano Gazzè, Bart Geboers, Natalie George, G Georgiadis, Daniel Gerber, Michelle Gerstman, Sammy Isabella Gharbieh, Samrad Ghavimi, Surjyendu Ghosh, Gianluca Giannarini, Philipp Gild, Inderbir Gill, Robyn Gillies, Ed Girgis, Nicola Giudici, Maximilian Glienke, Sidney Glina, Sanjith Gnanappiragasam, Jeremy Goad, Benjamin YS Goh, Hebe Gomes, Fernando Gómez, Seraina Gomez, Juan Gómez Rivas, Julio Cesar Gonzalez Brambila, Paavan Gorur, Vijaya Gottumukkala, Christopher Goßler, Niels Graafland, Arthur Grabsky, Markus Graefen, Michelle Anja Helene Graemiger, Matthieu Gratton, Christian Gratzke, Carmen Gravina, Isabella Greco, Gerard Grobler, Tobias Gross, Nico C Grossmann, Antonio Andrea Grosso, Robert große Siemer, Camilla Marisa Grunewald, Salvatore Guaglianone, Alessandro Guercio, A. Guerra, Ana Guijarro Cascales, Bertrand Guillonneau, Anil Gupta, Nishkarsh Gupta, Raghav Gupta, Georgi Guruli, Johannes Gussone, Andres Felipe Gutierrez Rojas, Thomas Hachenberg, Ibrahim Hacibey, Boris Hadaschik, Marios Hadjipavlou, Marinus J. Hagens, Luciano Gabriel Haiquel, Malik Hajidae, Oliver Hakenberg, Oliver Hald, Agus Rizal Hamid, Peter Hammerer, Karl Hampl, Analena Elisa Handke, Caelán Max Haney, Peter Hanna, Sajid Azeem Haque, Laurence Milner Harewood, Nina Natascha Harke, Matthew Harper, Julia Wenonah Hartl, Tim Hartl, Lorine Häuser, Dickon Hayne, Matthias M. Heck, Miriam Hegemann, Axel Heidenreich, Flavio Lobo Helwein, Sabrine Hemmes, Kees Hendricksen, Martin Johannes Peter Henning, Patrick Joseph Hensley, Thomas Hermanns, Thomas Hermans, Nicolas Hermieu, David J. Hernandez, Virginia Hernández Cañas, Roman Herout, José Antonio Herranz Yagüe, Jonathan Hiller, Maren Himmler, Lucy Elizabeth Hindle, Stephan Hintermeier, Benedikt Hoeh, Marinka Hoek, Jean Kuc Hoepffner, Stefan Hof, Dominik Charles Högger, Angela Holmes, Sebastian Homann, Anne Hong, Milan Hora, Satoshi Horii, Wolfgang Horninger, Ben Horsburgh, Hong Hong Huang, Sean Huang, Philipp Huber, Isabel Hughes, Crespin Hugo, Lau Yie Hui, Clara Humke, Dorjan Huqi, David Hutchinson, Stefan Huybrechts, Youssef Ibrahim, Paiboon Iemsupakkul, Roberta Ientile, Yu Imai, Ahmed Imran, Mehmet Reşat Inal, Gallus B. Ineichen, Brant A. Inman, Mariachiara Ippolito, Joseph Ischia, Claire Ishak, Samina Ismail, Kittikan Itthisan, Ranganathan Iyer, Kouji Izumi, Wolfgang Jaeger, Jitendra Jagtap, Salman Jamil, Nicholas Jansen, Jonas Jarczyk, Edouard Jarry, Saqib Javed, Claudio Jeldres, Anong Jenia, Pocharapong Jenjitranant, Stephan Jenzer, Pascal Jerney, Estíbaliz Jiménez-Alcaide, Varagorn Jirajan, Lydia Johns Putra, Ricardo Jordão Duarte, Manu Joris, Pierre Joubert, Patrick Juliebø-Jones, Mona Kafka, Yamini Kailash, Arveen Kalapara, Sebastian Kälble, Christos E Kalfountzos, Lia Mayumi Kubota Kamada, Gokul Vignesh Kandaswamy, Jada Kapoor, Guram Karazanashvili, Nino Karazanashvili, Sunaina Tejpal Karna, Alexander Kaserer, Cordelia Kaspar, Wassim Kassouf, Madlen Marie Elisabeth Kasten, Stamatios Katsimperis, Andreas Katsios, Christopher Kauffman Ortega, Ernest Kaufmann, Tatsushi Kawada, Jamie Kearsley, Nicola Keller, Brian Daniel Kelly, Timothy Kenyon-Smith, Zoe Keon-Cohen, Claudia Kesch, Ali Ahsan Khalid, Azhar Khan, Mohamed Shamim Khan, Sardar MJ Khan, Shahid Khan, Hau Chun Khoo, Kittinut Kijvikai, Lawrence H. Kim, Ned Kinnear, Bernhard Kiss, Marina Klaassen, Tobias Klatte, Sandra Kleinhans, Jakob Klemm, Stefanie Klenke, Claudia Béatrice Kluge, Hyla Kluyts, Thomas Knoll, Mara Koelker, Olivia Köhle, Sandeep Kondisetty, Badrinath Konety, Christopher Ho Chee Kong, Saranras Kongthanomrux, Mark Vincent Koning, Nick Julius Koning, Kevin Koo, Fernando Korkes, Philipp Korn, Jan-Wiebe Korstanje, Ersin Köseoğlu, Anthony Koupparis, Viktor Kovacik, Karl-Friedrich Kowalewski, Emefa Kporku, Mario Wolfgang Kramer, Zisis Kratiras, Philipp Krause, Alexander Kretschmer, Maximilian C Kriegmair, Jamie Krishnan, Sucha Kritsing, Bernard D Krüger, Sara Krüger, Albert Patryk Krzak, Katharina Kuhlencord, Girish Kulkarni, Jagdeesh N Kulkarni, Meghana Kulkarni, Francesca Kum, Naveen Kumar, Umut Kutukoglu, Jia-Lun Kwok, Mathew Yamoah Kyei, Emilia Labocetta, Louis Lacombe, Christian Ladurner, Michael Ladurner, Abdullah Laher, Alexander Laird, Alastair D Lamb, Glenn Lamers, Lucas Landen, Daniel Lane, Jasmine Lane, Bjoern Lange, Laura Langendries, Marcelo Langer Wroclawski, Francisca Larenas Huguet, Tim Larner, Javier D Lasala, Noémie Lautenbach, Terence Yu Xi Law, Nathan Lawrentschuk, Mahmoud Laymon, Lazaros Lazarou, Byron Lee, Elaine Lee, Hsiang Ying Lee, Charoen Leenanupunth, Maximilian Lenhart, Riccardo Leni, Costantino Leonardo, Jeffrey Leow, Scott Leslie, Tom Leslie, David Ka-Wai Leung, Nikolaos Liakos, Evangelos Liatsikos, Leslie Claire Licari, Fredrik Liedberg, Elena Lievore, Giovanni Liguori, Kheng Sit Lim, Kylie Yen-Yi Lim, Vera Lim, Pasin Limudomporn, Borje Ljungberg, Carlos Llorente Abarca, Alexander Lloyd, Niyati Lobo, Michele Lodde, Lukas M Löffel, A Lopatkin, Francisco Lopez, José Agustín López González, Manuel López-Baamonde, Björn Löppenberg, Yair Lotan, Raul Loures, Ilaria Lucca, Lorenzo Luciani, Evelyn Luggauer, Supanut Lumbiganon, Per-Olof Lundgren, Lukas Lunger, Benjamin Lyttwin, Christoph Lyttwin, Samson Ma, Moritz Maas, Findlay Macaskill, Alastair Roderick Macdonald, Petr Macek, Finlay Macneil, Marta Magaldi Mendaña, Kiran Mahendru, Osama Mahmoud, Elisabeth Maier, Julien Maillard, Edward Mains, Maroš Majer, David Mak, Sachin Malde, Rafaela Malinaric, David Mally, Mariangela Mancini, Sanjiban Mandal, Qusay Mandoorah, Lukas Manka, Todd Manning, Roy Mano, Paul Manohar, Ioannis Manolitsis, Guglielmo Mantica, Debora Maravigna, Gautier Marcq, Paramananthan Mariappan, Bartlomiej Markowski, George Martin, Paula Masgoret Olsina, Viraj A. Master, Riccardo Mastroianni, Alessia Mattei, Ravimohan Suryanarayan Mavuduru, Wolfgang Mayer, Roman Mayr, Giorgio Mazzon, Giovanni Mazzucato, Jonathan McCafferty, Steve McCombie, Arthur McPhee, Thibault Meert, Nicholas Mehan, Richard P Meijer, Valentin Meissner, Xiaosong Meng, Hanna Saskia Menold, David Merrilees, Laura Susanna Mertens, Tobias Metzger, Christian P Meyer, Luca-Marie Meyer, Salvatore Micali, Luca Miceli, Stefanie Michel, Eric R. Midenberg, Nicole Milenkovski, Maria Carmen Mir, Gus Miranda, Giovanni Misseri, Mohamed Ashraf Mohamed Daud, Taofiq Olayinka Mohammed, Felix Moltzahn, Romy Mondschein, Daniel Moon, Nelson Morales Palacios, Kim L. Moretti, Stefano Moretto, Keiichiro Mori, Ahmed Mosbah, Marco Moschini, Dimitrios Moschonas, Marcio Covas Moschovas, Basil Francis Moss, Hadi Mostafaei, Angelo Mottaran, Nicolas Mottet, Alexandre Mottrie, Uwais Mufti, Clancy Mulholland, Caspar Müller, Mareike Muny, Srikanth Murali-Krishnan, Declan Murphy, Prithvi Murthy, Shingai Mutambirwa, Antonio Nacchia, Mehwash Nadeem, Rajesh Nair, Tohru Nakagawa, Benjamin Namdarian, Shintaro Narita, Gregory Nason, Richard Naspro, Arjun Nathan, Neema Navai, Ludovica Navarra, Faisal Nawaz, Arvind Nayak, Rishi Nayyar, Justin Nazareth, Syed Muhammad Nazim, Alexander L Nesbitt, Martin Neukirchen, Elena Neumann, Kay-Seong Ngoo, Michele Nicolazzini, Günter Niegisch, Julia Niegisch, Malin Nientiedt, Jonathan Noel, José Ignacio Nolazco, Joachim Noldus, Anne Nordstrom, Peter H. Norman, Briony Norris, Valentina Norz, Mahmoud Nosseir, Marco Notarfrancesco, Adam Novák, Vladimir Novotny, Duarte Silva Nuno Dias, Jonathan O’Brien, Michael O’Callaghan, David Peter Obert, Mario Ochoa Arvizo, Pascal Oechslin, Carlos Henrique Franco Oliveira, M’Baya Olivier, Valerio Olivieri, Abisola Oliyide, Akinlolu Oluwole-Ojo, Kawa Omar, Richard Ondrejcek, Chloe She Hui Ong, Sean Ong, Teng Aik Ong, Luca Ongaro, Desire Onwochei, Jason Ooi, Immanuel Augustin Oppolzer, Nasir Orakzai, Flávio V. Ordones, Luca Orecchia, Luis Enrique Ortega Polledo, Christophe Orye, Yassar Osman, Hugo Otaola Arca, Idir Ouzaid, Adetokunbo Owolabi, Özlem Özkalaycı, Sarah O’Neill, Pia Paffenholz, Toby Page, Konstantinos Pagonis, Salvatore M. Palermo, Maximilian Pallauf, Carlotta Palumbo, Henry Yen-Cheng Pan, Edward Jian Xu Pang, Kan Panyapinitnugoon, Nathan Papa, Rocco Papalia, Dimitrios Papanikolaou, Riccardo Papetti, Aditya Parikh, Luke Parkes, Michael Stephan Pärli, Brian Andrew Parsons, Alexandre Dib Partezani, Giuseppe Pascarella, Caio Pasquali, Francesco Passaro, Antonio Luigi Pastore, Manish I. Patel, Vipul Patel, Dattatraya H. Patil, Giulio Patruno, Kevin Thomas Pattberg, Nicola Pavan, Francesco Pellegrino, Sisto Perdonà, Lucas Antonio Pereira do Nascimento, Sachin Perera, Nathan Perlis, Angelo Peroni, Ophélie Perrot, Arsalan Pervaiz, Dario Pesce, Andrea Pescuma, Angelis Peteinaris, Lukas Peter, Justin S Peters, Drew Phillips, Catherine Phoenix, Alberto Piana, Andrea Piasentin, John Piedad, Phillip M. Pierorazio, Amelia Pietropaolo, Geraldine Pignot, Alberto Piller, Narutsama Pimpanit, Ugo Pinar, Nisha Pindoria, Sohan Zane Pinto, Federico Piramide, Adele Piro, Giacomo Maria Pirola, Surawach Piyawannarat, Guillaume Ploussard, Keyuri Popat, Francesco Porpiglia, James Porter, Philip Posdzich, Randi Marisa Pose, Thomas Poulton, Cédric Poyet, Benjamin Pradere, Kris B. Prado, Felix Preisser, Yaamini Premakumar, Gabriele Presti, Flavia Proietti, Gideon Ptasznik, Ignacio Puche-Sanz, Felipe Guimarães Pugliesi, Stefano Puliatti, Juliane Putz, Armin Pycha, Kirby R. Qin, Liang G. Qu, Leonardo Quarta, Harris Hassan Qureshi, Robert S. Radcliffe, Jan Philipp Radtke, Mauro Ragonese, Fakhri Rahman, Nicholas Raison, Jay D. Raman, Vinicius Ramos Machado, Pradeep Rao, Ranjit Rao, Jens Rassweiler, Marie-Claire Rassweiler-Seyfried, Benjamin Raunecker, Steffen Rausch, Pedro Recabal-Guiraldes, Enrique Redondo Gonzalez, Cristina Redondo Redondo, Abhishek Kumar Reekhaye, Fairleigh Reeves, Ivo B. Regli, Alina Reicherz, Moritz J Reike, Jennifer R Reilly, Philipp Reimold, Giuseppe Reitano, Isabell Rektorik, Luigi Maria Remore, Johan Renaldo, Peera Rhunsiri, María Riaza Montes, Maria J. Ribal, Rafael Ribeiro Meduna, Patrick O. Richard, Bernhard Riedel, Lorena Rifa, Joanneke B. Ringia, Michael Rink, Benjamin T. Ristau, Michele Rizzo, Severin Rodler, Alejandro Rodriguez, Olivia Rodriguez, Natali Rodriguez Peñaranda, Lara Rodriguez-Sanchez, Moises Elias Rodríguez-Socarrás, Florian Roghmann, Georg Rohe, Giulio Luca Rosboch, Bernd Rosenhammer, Marta Rossanese, Julian Rössler, Beat Roth, Mathieu Roumigué, Morgan Roupret, Edward Rowe, Tamas Rozsos, Manapol Rujithamkul, Julian Runge, Marco-Christopher Rupp, Michele Russo, Stephen Ruthven, Vera C. Rutten, João Jorge Saab Filho, Arun Sahai, Sebastian Daniel Sahli, Shady Saikali, A Sanchez, Rafael Sanchez Salas, Noah Sandel, Camilo Sandoval-Herrera, Sama Sappayasarn, Sutthirat Sarawong, Prassannah Satasivam, Niranjan Sathianathen, Richard Savdie, Ziv Savin, Roberto Mario Scarpa, Matthijs J. Scheltema, Robert Schier, Manuel Schilling, Martin Schlaepfer, Benjamin Schmeusser, Hans-Peter Schmid, Frederick Schneider, Daniel Schöndorf, Martin Schostak, Belinda Schramm, Hans Harold Schudel, Gerald Schulz, Christian Schwentner, Helena Scott, Simone Scuderi, Benjamin Sebastian, Fernando Secin, Kulthe Seetharam Bhat, Kanesh Kumaran Seevalingam, Elena Seguí-Moya, Roland Seiler, Cagri Akin Sekerci, Timur Sellmann, Shomik Sengupta, Amlesh Seth, Kapil Sethi, Tarapon Setthawong, John Sfakianos, Jay B Shah, Shagun Bhatia Shah, Shahzad Shah, Asad Shahzad Hasan, Shahrokh F Shariat, Aditya Prakash Sharma, Apoorva Shastri, Greg Shaw, Matin Sheriff, Je Song Shin, Masaki Shiota, Asaf Shvero, Mookdarat Siantong, Karl-Dietrich Sievert, August Sigle, Neha Sihra, Luigi Silvestri, Giuseppe Simone, Ajay Singh, Jas Singh, Shrawan K. Singh, Harshit Singla, Nirmish Singla, Leandro Siragusa, Kun Sirisopana, Eila C. Skinner, Daron Smith, Oliver Smith, Peter Soeding, Charlotte Soenens, Timo Soeterik, Sohan Lal Solanki, Christopher Soliman, Bhaskar Somani, Marcus Sondermann, Naeem Soomro, Gianluca Spena, Claudia Fede Spicchiale, Philippe Spiess, Victor Srougi, Fabian P. Stangl, Pieter Jan Steelant, Philipp G. Stein, Daniel Steiner, Giuseppe Stella, Arnulf Stenzl, Christian Stief, Jordi Stira, Elena Stojkova Gafner, Konrad Stephan Jakob Straub, Kay Stricker, Kevin Stritt, Johann Stuby, Moe Thiha Swe, Firtantyo Adi Syahputra, Madiha Syed, Diaa-Eldin Taha, Yanni Tan, Marco Tanello, Vincent Tang, Songyos Tangmesang, Simon Tanguay, Manisa Tangvipattanapong, Yiloren Tanidir, Stefano Tappero, Melina Tatalias, Cesar Antonio Tavares Da Rocha, Francesco Tedesco, Bernardo Lobão Afonso Teixeira, Riccardo Tellini, Patrick Teloken, Catherine Temelcos, Heidi Tempest, Beatriz Tena, N Tenke, Pierre Tennstedt, Jeremy Yuen-Chun Teoh, Clara Tesche, Antonio Testa, Stefanie Thaler, George Thalmann, Nutdanai Thammasaksit, Thanathorn Thampravit, Dinh Phuong Thao, Shankaran Thevarajah, Benjamin Thomas, Christian Thomas, Ramesh Thurairaja, Marco Ticonosco, Ho Yee Tiong, Ko Ko Zayar Toe, Musliu Adetola Tolani, Raymond Tong, Werner Tonnetzer, Giulia Torregiani, Fabio Traunero, Peter Trebo, Emanuela Trenti, Monica Trivedi, Richard Truse, Dimitar Mihaylov Tsarov, Arman Tsaturyan, Stephan Tschirdewahn, Christopher Tschung, Andrea Tubaro, Gabriele Tuderti, Karl Tully, Rafal Turo, Filippo Maria Turri, Alexander Tzabazis, Lazaros Tzelves, Mohsin Uddin, Martin H. Umbehr, Vanessa Upadek, Ronald James Urry, Massimo Valerio, Anthony Van Baelen, Michael R. van Balken, Eva Van Bos, Siska van Bruwaene, Astrid Van Daela, Roderick van den Bergh, Antoine G van der Heijden, Andre van der Merwe, Henk G. van der Poel, Koene van der Sloot, Danielle Charlotte Van Diepen, Charles Van Praet, Bas WG van Rhijn, Stijn Van Vugt, Joris Vangeneugden, Matthias Vanneste, Mihai Dorin Vartolomei, Nikhil Vasdev, Antonio Vavallo, Francesca Vedovo, Hans Veerman, Ian Vela, Marina Vendrell Jordà, Pieter Verleyen, Lodewikus P. Vermeulen, Malte W. Vetterlein, Ralf Veys, Fabio Carvalho Vicentini, Alvaro Vidal-Faune, Matheus Vieira dos Santos, Silvia Viganò, Antoni Vilaseca, Gianluca Villa, Wit Viseshsindh, Michiel Vlaming, Jonathan Vollemaere, Alessandro Volpe, Yannic Volz, Maximilian Ferry von Bargen, Markus von Deimling, Thilo Caspar von Groote, Friedrich Von Rundstedt, Thomas Adrian von Rütte, Franz Von Stauffenberg, Charlotte S. Voskuilen, Srinivas Vourganti, Florian Wagenlehner, Aziz Wajahat, Anjana Sagar Wajekar, Margarete Teresa Walach, Anna Lucy Walsh, Thomas J. Walton, Jochen Walz, William Wang, Ziting Wang, Tomofumi Watanabe, Chinnawat Wattana, Laurent-Michel Wattier, Kittisak Weerapolchai, Mirja Wegge, Christopher Weight, Laurence Weinberg, Sylvia Weis, Raphael Weiss, Johann Wendler, Madeleine Wengi, Mike Wenzel, Samuel Weprin, Lukas Alex Wernli, Frederik Wessels, Niklas Westhoff, Robin Weston, Katy-Louise Whelan, Lucas R. Wiegand, Laura Wiemer, Carl J. Wijburg, Isabella Williams, Simon Williams, Susan Willis, Alexander Winter, Oliver Wiseman, Esther Mk Wit, JAlfred Witjes, Solomon Woldu, Chris Ho-Ming Wong, Piyapong Wongjittraporn, Henry Woo, Dixon Teck Sing Woon, Thomas Stefan Wort, Fiona Wu, Michael Wu, Wen-Jeng Wu, Patrick Y. Wuethrich, Christoph Würnschimmel, Yves Wyss, Jennifer Xu, Evanguelos Xylinas, Takafumi Yanagisawa, Tet Yap, Omid Yassaie, John Yaxley, Hsin-Chih Yeh, Wie Sein Yeoh, Yuyi Yeow, Leubet Yirga, Daniel ZP Yong, Joseph Zabell, Zainal Abiddin Zainal Adwin, Carlo Zaraca, Stefano Zaramella, Homi Zargar, Fabio Zattoni, Alan Javier Zazú, Michel Zbinden, Tereza Zdobinska, Pascal Zehnder, Claudia Zelger, Barbora Zemlickova, Logan Zemp, Kenji Zennami, Yuhao Zhang, Valentin Zumstein

**Affiliations:** 1 Department of Urology, UK-OWL, Campus Klinikum Lippe, Detmold, Germany; 2 Guy’s and St Thomas’ NHS Foundation Trust, London, United Kingdom; 3 Division of Urology, University of the Witwatersrand, Johannesburg, South Africa; 4 Mediteranean Private Hospital, Limasol, Cyprus; 5 University Hospital of Patra, Greece; 6 Division of Surgical Oncology, Afe Babalola University Multisystem Hospital, Ado-Ekiti, Nigeria; 7 Department of Anaesthesiology and Pain Medicine, Inselspital Bern, University Hospital of Berne, Bern, Switzerland; 8 Department of Urology, Luzerner Kantonsspital, University of Lucerne, Lucerne, Switzerland; 9 Jupiter Hospital, Mumbai, India; 10 Department of Urology, The Royal Melbourne Hospital, Parkville, Australia; 11 Department of Anaesthesia, Critical Care and Pain, Tata Memorial Hospital, Homi Bhabha National Institute, Mumbai, India; 12 Urology Unit, Azienda Ospedaliero Universitaria delle Marche, Università Politecnica delle Marche, Ancona, Italy; 13 Department of Urology, Seth Gordhandas Sunderdas (GS) Medical College and King Edward Memorial (KEM) Hospital, Mumbai, India; 14 Urología, Hospital Universitario Puerto Real, Cadiz, Spain; 15 Sheikh Shakhbout Medical City and Medical School, Sheikh Khalifa University, Abu Dhabi; 16 Department of Urology, Solothurner Spitäler AG, Kantonsspital Olten and Bürgerspital Solothurn, Switzerland; 17 Institute of Anaesthesiology, University Hospital of Zurich, Zurich, Switzerland; 18 Department of Urology, Faculty of Medicine, Çukurova University, Adana, Turkey; 19 Bristol Urological Institute, North Bristol NHS Foundation Trust, Bristol, United Kingdom; 20 Department of Urology, University Hospital and Medical Faculty, Heinrich-Heine-University, Düsseldorf, Germany; 21 Department of Urology, Crouse Hospital, Syracuse, NY, United States of America; 22 Division of Urology, UConn Health, Farmington, CT, United States of America; 23 Department of Anaesthesia and ICU, Modibbo Adama University Teaching Hospital, Yola, Nigeria; 24 Urology Department, Urology and Nephrology Center, Mansoura University Faculty of Medicine, Mansoura, Egypt; 25 Department of Urologic Sciences, University of British Columbia, Vancouver, Canada; 26 Department of Urology, Charité University Hospital, Berlin, Germany; 27 University of Jeddah, Jeddah, Saudi Arabia; 28 Department of Urology, Kreiskrankenhaus Reutlingen, Germany; 29 Urology Department, AZ Maria Middelares, Ghent, Belgium; 30 IRCCS Candiolo Cancer Institute, Candiolo, Italy; 31 Klinik für Urologie, Universitätsklinikum Jena, Jena, Germany; 32 Department of Urology, IRCCS “Regina Elena” National Cancer Institute, Rome, Italy; 33 Division of Urology, Department of Surgery, Faculty of Medicine Ramathibodi Hospital, Mahidol University, Bangkok, Thailand; 34 The James Cook University Hospital, South Tees Hospitals NHS Foundation Trust, Middlesbrough, United Kingdom; 35 Department of Urology, Hospital Universitario de Fuenlabrada, Madrid, Spain; 36 Section of Urology , Department of Surgery, The Aga Khan University, Karachi, Pakistan; 37 Belfast City Hospital, Northern Ireland, United Kingdom; 38 Department of Urology, State Hospital Bregenz, Bregenz, Austria; 39 Department of Urology, Eberhard-Karls-University Tübingen, Germany; 40 Department of Anaesthesiology, University Hospital Essen, Essen, Germany; 41 Glickman Urological Institute, Cleveland Clinic, Cleveland, OH, United States of America; 42 Department of Urology, München Klinik Bogenhausen, Munich, Germany; 43 Department of Urology, Teaching Hospital Motol, 2nd Faculty of Medicine, Charles University, Prague, Czech Republic; 44 Klinik für Urologie, Kantonsspital St. Gallen, St. Gallen, Switzerland; 45 University of Tartu, Tartu, Estonia; 46 Instituto de Assistência Médica ao Servidor Público Estadual, São Paulo, Brazil; 47 Department of Urology, Mount Sinai Icahn School of Medicine, Elmhurst/Queens Hospitals, NY, United States of America; 48 Department of Urology, University Hospitals Leuven, Leuven, Belgium; 49 Department of Urology, Marien Hospital Herne, Universitätsklinikum der Ruhr-Universität Bochum, Bochum, Germany; 50 Unit of Urology/Division of Oncology, Urological Research Institute (URI), IRCCS San Raffaele Scientific Institute, Milan, Italy; 51 Sir Charles Gardiner Hospital, Nedlands, Australia; 52 London North West University Healthcare NHS Foundation Trust, Harrow, United Kingdom; 53 Department of Anaesthesiology, University Medical Center Hamburg-Eppendorf, Hamburg, Germany; 54 Department of Urology, Singapore General Hospital, Singapore; 55 2nd Department of Urology, National and Kapodistrian University of Athens, Sismanogleio General Hospital, Athens, Greece; 56 Division of Urology, Geneva University Hospitals, Geneve, Switzerland; 57 Department of Urology, University Hospital Pilsen, Czech Republic; 58 Department pf Urology, University of Modena and Reggio Emilia, Modena, Italy; 59 Urology Department, Claude Huriez Hospital, CHU Lille, Lille, France; 60 Institute of Intensive Care, University Hospital of Zurich, Zurich, Switzerland; 61 Klinik für Urologie, Universitätsklinikum Halle (Saale), Halle, Germany; 62 Centro Hospitalar e Universitário de Lisboa Central, Lisboa, Portugal; 63 Department of Urology, ERN eUROGEN accredited center, Ghent University Hospital, Ghent, Belgium; 64 Department of Human Structure and Repair, Faculty of Medicine and Health Sciences, Ghent University, Ghent, Belgium; 65 Department of Urology, Netherlands Cancer Institute, Amsterdam, The Netherlands; 66 Department of Urology, University of Modena and Reggio Emilia, Modena, Italy; 67 San Carlo di Nancy Hospital, Rome, Italy; 68 Urologie am Stephanshorn, St. Gallen, Switzerland; 69 Urological Competence Centre for Rehabilitation, Klinik Wildetal, Kliniken-Hartenstein, Bad Wildungen, Germany; 70 Department of Oncology, Inselspital Bern, Bern, Switzerland; 71 Division of Urology, Centre Hospitalier de l’Université de Montréal, Montreal, Canada; 72 Department of Urology, University of Verona, AOUI Azienda Ospedaliera Universitaria Integrata Verona, Verona, Italy; 73 Department of Urology, European Institute of Oncology (IEO) IRCCS, Milan, Italy; 74 Department of Urology, University Hospital of Zurich, Zurich, Switzerland; 75 Urologie St. Anna, Lucerne, Switzerland; 76 Department of Urology, Western Health, Footscray, Melbourne, Australia; 77 Department of Urology, NHS Lothian, Edinburgh, United Kingdom; 78 Department of Urology, Graduate School of Medical Sciences, Kyushu University, Japan; 79 Department of Urology, Monash Medical Centre, Melbourne, Australia; 80 Department of Anaesthesia and Intensive Care, “F. Tappeiner” Hospital, Merano, Italy; 81 Department of Urology, University Hospital Carl Gustav Carus, TU Dresden, Dresden, Germany; 82 Department of Anaesthesiology and Critical Care Medicine, University Medical Centre Mannheim, Medical Faculty Mannheim of the University of Heidelberg, Mannheim, Germany; 83 Department of Urology, University of Health Sciences, Antalya Training and Research Hospital, Antalya, Turkey; 84 Sapienza University of Rome, Rome, Italy; 85 Department of Urology, Austin Health, Melbourne, Australia; 86 Department of Urology, Azienda Sanitaria Universitaria Giuliano Isontina, Trieste, Italy; 87 Department of Urology, Erasmus MC Cancer Institute, Rotterdam, The Netherlands; 88 Department of Urology, University of Rostock, Rostock, Germany; 89 Servicio de Urología, Hospital Universitario de Getafe, Getafe, Spain; 90 Urology Unit, Lindenhof Hospital Bern, Switzerland; 91 Dipartimento di Anestesia, Rianimazione ed Emergenza-Urgenza, Fondazione IRCCS Ca’ Granda Ospedale Maggiore Policlinico, Milan, Italy; 92 Department of Anesthesiology, University Hospital Göttingen, Göttingen, Germany; 93 Department of Urology, Hesperia Hospital Modena, Modena, Italy; 94 AZORG Hospital, Aalst, Belgium; 95 The University of Texas MD Anderson Cancer Center, Houston, TX, United States of America; 96 Department of Urology, Mayo Clinic AZ, Phoenix, AZ, United States of America; 97 Department of Urology, LMU University Hospital, LMU Munich, Munich, Germany; 98 Department of Urology, Royal Prince Alfred Hospital, Syndrey, Australia; 99 Department of Anaesthesiology, Netherlands Cancer Institute, Amsterdam, The Netherlands; 100 Department of Urology, Medical University Vienna, Vienna, Austria; 101 Department of Urology, University Medical Center Hamburg-Eppendorf, Hamburg, Germany; 102 Urology Department, University Hospital of Tours, Tours, France; 103 Department of Anesthesiology and Intensive Care, University Hospital Rechts der Isar, Technical University of Munich, Munich, Germany; 104 Department of Urology, Caritas, St. Josef Medical Center, University of Regensburg, Regensburg, Germany; 105 Medway Maritime Hospital, Medway NHS Foundation Trust, Gillingham, United Kingdom; 106 University College Hospital London NHS Foundation Trust, London, United Kingdom; 107 Hospital General de La Palma, Santa Cruz de La Palma, Spain; 108 USC Institute of Urology and Catherine and Joseph Aresty Department of Urology, Keck School of Medicine, University of Southern California, Los Angeles, CA, United States of America; 109 Campus Biomedico University of Rome, Rome, Italy; 110 Department of Urology, London Bridge Hospital, London, United Kingdom; 111 Department of Urology, The Royal Marsden NHS Foundation Trust, London, United Kingdom; 112 Department of Anaesthesology, GZO Spital Wetzikon, Switzerland; 113 Hospital Municipal Brigadeiro, São Paulo, Brazil; 114 Department of Urology, Faculty of Medicine, Istanbul Medipol University, Istanbul, Turkey; 115 East Sussex Healthcare NHS Foundation Trust, Eastbourne, United Kingdom; 116 Cardiac Surgery ICU, Ospedale San Gerardo, Monza, Italy; 117 Unit of Oncologic Minimally-Invasive Urology and Andrology, Azienda Ospedaliero Universitaria Careggi, Florence, Italy; 118 Department of Experimental and Clinical Medicine, University of Florence, Florence, Italy; 119 Department of Urology, University Hospital of Parma, Parma, Italy; 120 Hôpital Tenon, Hôpitaux Universitaires de l’Est Parisien, APHP-Sorbonne Université, Paris, France; 121 Department of Urology, Koç University, Istanbul, Turkey; 122 Pontifical Catholic University of Rio Grande do Sul, Porto Alegre, Brazil; 123 University Medical Center Groningen, Groningen, The Netherlands; 124 Division of Urology, Department of Surgery, L’Institut Mutualiste Montsouris, Paris, France; 125 Department of Medicine, Unit of Anesthesia, Intensive Care and Pain Management, Campus Biomedico University of Rome, Rome, Italy; 126 University of Sheffield, Sheffield, United Kingdom; 127 Niguarda Hospital, Milan, Italy; 128 Policlinico Umberto I, Sapienza University of Rome, Rome, Italy; 129 ASL Abruzzo 1 Avezzano Sulmona L’Aquila, Italy; 130 Department of Anesthesia, Transplant and Surgical Intensive Care, Azienda Ospedaliero Universitaria delle Marche, Università Politecnica delle Marche, Ancona, Italy; 131 Department of Anaesthesiology, Koç University, Istanbul, Turkey; 132 Cleveland Clinic, OH, United States of America; 133 Universiti Malaya Medical Centre, Kuala Lumpur, Malaysia; 134 University of the Witwatersrand, Johannesburg, South Africa; 135 School of Medicine, University of Sydney, Sydney, Australia; 136 Warrington and Halton Teaching Hospitals NHS Foundation Trust, Warrington, United Kingdom; 137 Prince of Wales Hospital, The Chinese University of Hong Kong, Hong Kong; 138 Department of Urology, Lister Hospital, East and North Herts Teaching NHS Foundation Trust, Stevenage, United Kingdom; 139 Urology Center, Ambala, India; 140 Centre for Integrated Oncology (CIO) Düsseldorf, CIO Aachen-Bonn-Cologne-Düsseldorf, Düsseldorf, Germany; 141 Department of Urology, James Buchanan Brady Urological Institute, Johns Hopkins University School of Medicine, Baltimore, MD, United States of America; 142 St. Luke’s University Health Network, Fountain Hill, PA, United States of America; 143 Department of Urology, Kaohsiung Medical University Hospital, Kaohsiung Medical University, Kaohsiung, Taiwan; 144 Stockport NHS Foundation Trust, Stockport, United Kingdom; 145 Asian Institute of Nephrology and Urology, Hyderabad, India; 146 Thomas Jefferson University, Philadelphia, PA, United States of America; 147 Ospedale Sant’Andrea, Sapienza University of Rome, Rome, Italy; 148 Department of Intensive Care Medicine, Kantonsspital Aarau, Aarau, Switzerland; 149 Department of Anesthesia and Intensive Care, Hospital Universitario La Paz, Spain; 150 Department of Urology, Instituto Valenciano de Oncologia Fundacion, Valencia, Spain; 151 Oncological Urology, Veneto Institute of Oncology (IOV) IRCCS, Padua, Italy; 152 Department of Urology, Hospital of Bolzano (SABES-ASDAA), Bolzano-Bozen, Italy; 153 Servicio de Anestesiología y Reanimación, Hospital Clínic, Barcelona, Spain; 154 Sheffield Teaching Hospitals NHS Foundation Trust, Sheffield, United Kingdom; 155 Department of Urology, Istituto Nazionale Tumori IRCCS Fondazione G. Pascale, Naples, Italy; 156 Department of Precision Medicine in Medical, Surgical and Critical Care, University of Palermo, Palermo, Italy; 157 ASST Papa Giovanni XXIII, Bergamo, Italy; 158 University of Perugia, Italy; 159 Wesley Urology Clinic, The Wesley Hospital, Brisbane, Australia; 160 Department of Urology, University Hospital of Wales, Heath Park, NHS Wales, Cardiff, United Kingdom; 161 Oncological Urology, Veneto Institute of Oncology IOV - IRCCS, Padua, Italy; 162 Department of Urology, Policlinico Tor Vergata, University of Tor Vergata, Rome, Italy; 163 Memorial Sloan-Kettering Cancer Center, New York City, NY, United States of America; 164 Department of Diagnostics and Intervention, Urology and andrology, Umeå University, Umeå, Sweden; 165 Department of Urology, University Hospital Essen, Essen, Germany; 166 Department of Urology, AZ Maria Middelares, Ghent, Belgium; 167 Department of Urology, HELIOS University Hospital Wuppertal, Germany; 168 Toowoomba Urology, Toowoomba, Australia; 169 Department of Anaesthesia and Intensive Care, PGIMER, Chandigarh, India; 170 Ghent University, Ghent, Belgium; 171 Anesthesia and Intensive Care Unit, Mazzoni Hospital, Ascoli Piceno, Italy; 172 Urology, Hospital de Braga, Braga, Portugal; 173 Fundación Arturo López Pérez, Santiago, Chile; 174 University Hospitals Plymouth NHS Foundation Trust, Plymouth, United Kingdom; 175 Department of Urology, Ballarat Base Hospital (BBH), Ballarat, Australia; 176 Institut für Anästhesiologie, Stadtspital Zürich, Zurich, Switzerland; 177 Northwest Permanente, Portland, Oregon, OR, United States of America; 178 Department of Anaesthesia and Pain Management, The Royal Melbourne Hospital, Parkville, Australia; 179 Università degli Studi di Sassari, Sassari, Italy; 180 Division of Urology, Department of Surgical Sciences, Faculty of Medicine and Health Sciences, Stellenbosch University, Stellenbosch, South Africa; 181 Department of Urology, Kent and Canterbury Hospital, East Kent Hospitals University, Canterbury, United Kingdom; 182 Department of Urology, Royal Surrey County Hospital, Guildford, United Kingdom; 183 Department of Urology and Urological Surgery, University Medical Center Mannheim, Medical Faculty Mannheim of Heidelberg University, Mannheim, Germany; 184 University of Health Sciences, Diskapi Yildirim Beyazit Training and Research Hospital, Ankara, Turkey; 185 Department of Andrology, Faculty of Medicine, Cairo University, Giza, Egypt; 186 Department of Anesthesiology, University Hospital and Medical Faculty, Heinrich-Heine-University, Düsseldorf, Germany; 187 Department of Surgery, Division of Urology, University Health Network - Princess Margaret Cancer Centre, University of Toronto, Canada; 188 Department of Anaesthesiology, Erasmus MC Cancer Institute, Rotterdam, The Netherlands; 189 Azienda Ospedaliero Universitaria Parma, Parma, Italy; 190 Darent Valley Hospital, Dartford and Gravesham NHS Foundation Trust, Dartford, United Kingdom; 191 Department of Development and Regenerations, K.U.Leuven, Leuven, Belgium; 192 Martini-Klinik Prostate Cancer Centre, University Hospital Hamburg-Eppendorf, Hamburg, Germany; 193 Hannover Medical School, Hannover, Germany; 194 Department of Urology, Unidade Local de Saúde de Matosinhos, Matosinhos, Portugal; 195 Department of Anaesthesia, Austin Health, Melbourne, Australia; 196 Department of Clinical and Experimental Medicine, Urologic Section, University of Messina, Messina, Italy; 197 Department of Oncology, Urologic Section, AOU G. Martino, Messina, Italy; 198 Hirslanden Klinik Cham, Cham, Switzerland; 199 Urology Unit, Magna Graecia University of Catanzaro, Catanzaro, Italy; 200 Royal North Shore Hospital, University of Sydney, Sydney, Australia; 201 Department of Urology, Westmoreland Street Hospital, UCLH NHS Foundation Trust, London, United Kingdom; 202 Anaesthesia Department, NHS Grampian, Aberdeen, United Kingdom; 203 Oncology Research program, CHU de Québec-Université Laval Research center and Cancer Research Center of Université Laval, Québec, QC, Canada; 204 Department of Surgery, Faculty of Medicine, Université Laval, Québec, QC, Canada; 205 Department of Urology, Slovak Medical University, Roosevelt Teaching Hospital, Banska Bystrica, Slovakia; 206 University of São Paulo School of Medicine, São Paulo, Brazil; 207 Department of Anaesthesiology, University Hospital Tübingen, Germany; 208 Cabrini Health, Malvern, Australia; 209 Urology Unit, Camposampiero Civil Hospital, Padua, Italy; 210 Division of Urology/Urooncology, Department of Surgery. School of Medicine, Universidad del Valle. Cali, Colombia; 211 All India Institute of Medical Sciences, New Delhi, India; 212 Garvan Institute of Medical Research, Darlinghurst, Australia; 213 Peter MacCallum Cancer Centre, Melbourne, Australia; 214 Santa Maria della Misericordia University Hospital, Udine, Italy; 215 Department of Urology, Spitalzentrum Biel, Biel, Switzerland; 216 Department of Urology, Faculty of Medicine, University of Freiburg Medical Center, Freiburg, Germany; 217 Department of Urology, Centro Universitário FMABC; 218 King’s College Hospital NHS Foundation Trust, London, United Kingdom; 219 Department of Urology, St Vincent’s Hospital, Melbourne, Australia; 220 Department of Urology, National University Hospital (NUH), Singapore; 221 Department of Urology, Instituto Cirugía Urológica Avanzada (ICUA), Madrid, Spain; 222 Servicio de Urología, Hospital Clínico San Carlos, Madrid, Spain; 223 Izmirlian Medical Center, Yerevan, Armenia; 224 Réseau hospitalier neuchâtelois, Neuchâtel, Switzerland; 225 Die Berner Urologen AG, Bern, Switzerland; 226 Urology Department, Hospital Universitario Fundación Alcorcón, Madrid, Spain; 227 Karolinska University Hospital, Stockholm, Sweden; 228 Department of Onco-Anesthesiology and Palliative Medicine, All India Institute of Medical Sciences, New Delhi, India; 229 VCU Health, Virgina Commonwealth University, Richmond, VA, United States of America; 230 Hospital Universitario San Ignacio, Bogotá, Colombia; 231 Department of Urology, Basaksehir Çam and Sakura City Hospital, Istanbul, Turkey; 232 Department of Urology, Cipto Mangunkusumo Hospital, Jakarta, Indonesia; 233 Department of Urology, Braunschweig Municipal Hospital, Germany; 234 Department of Anaesthesiology, Spitalzentrum Biel, Biel, Switzerland; 235 Department of Urology, University of Leipzig, Leipzig, Germany; 236 Department of Urology, University of Minnesota, MN, United States of America; 237 Epworth Healthcare, Melbourne, Australia; 238 Klinik für Anästhesiologie, operative Intensivmedizin, Schmerz- und Palliativmedizin, Marien Hospital Herne, Universitätsklinikum der Ruhr-Universität Bochum, Bochum, Germany; 239 Department of Urology, Ruhr-University Bochum, Marien Hospital Herne, Herne, Germany; 240 UWA Medical School, University of Western Australia, Perth, Australia; 241 Department of Urology, University Hospital Rechts der Isar, Technical University of Munich, Munich, Germany; 242 Urology, Faculty of Medicine, University of Augsburg, Augsburg, Germany; 243 Department of Urology, University Hospital of Cologne, Cologne, Germany; 244 Federal University of Santa Catarina, Brazil; 245 Klinik für Urologie, Universitätsklinikum Schleswig-Holstein, Campus Lübeck, Luebeck, Germany; 246 Department of Urology, University of Kentucky College of Medicine, Lexington, KY, the United States of America; 247 Department of Urology, Bichat-Claude Bernard Hospital, APHP, Paris Cité University, Paris, France; 248 University of South Florida, Tampa, FL, the United States of America; 249 Diakonieklinikum Stuttgart, Stuttgart, Germany; 250 Department of Urology, University Hospital Frankfurt, Goethe University Frankfurt am Main, Frankfurt am Main, Germany; 251 University of Pretoria, Pretoria, South Africa; 252 Urology Department, Clinique Saint Augustin, Bordeaux, France; 253 Department of Urological Surgery, Barwon Health, University Hospital Geelong, Geelong, Australia; 254 Klinikum Dortmund, Dortmund, Germany; 255 Okayama University Hospital, Okayama, Japan; 256 Epsom and St Helier University Hospitals, NHS Foundation Trust, United Kingdom; 257 Department of Anaesthesiology, Tan Tock Seng Hospital, Singapore; 258 Urology Department, Central Hospital of Bolyano, Italy; 259 Department of Urology, The Jikei University School of Medicine, Tokyo, Japan; 260 Department of Urology, Inselspital Bern, University Hospital of Berne, Bern, Switzerland; 261 Prostate and Urologic Cancer Center, Duke Cancer Institute, Durham, NC, United States; 262 Department of Anaesthesiology, Western Health, Footscray, Melbourne, Australia; 263 Department of Anaesthesiology, The Aga Khan University, Karachi, Pakistan; 264 Chaophraya Yommaraj Hospital, Suphanburi, Thailand; 265 Kanazawa University Hospital, Kanazawa, Japan; 266 Department of Urology, University Medical Center, Mainz, Germany; 267 Sir H. N. Reliance Foundation Hospital and Research Centre, Mumbai, India; 268 Bakhtawar Amin Trust Teaching Hospital, Multan, Pakistan; 269 Centre Hospitalier Universitaire de Sherbrooke, Université de Sherbrooke, Sherbrooke, Canada; 270 Hospital das Clinicas, São Paulo University, São Paulo, Brazil; 271 Haukeland University Hospital, Bergen, Norway; 272 Department of Urology, Medical University of Innsbruck, Austria; 273 Bendigo Health, Bendigo, Australia; 274 General Hospital of Larissa Koutlimpaneio and Triantafylleio, Larissa, Greece; 275 Hospital do Servidor Público Estadual de São Paulo, São Paulo, Brazil; 276 Swansea Bay University Health Board, NHS Wales, Port Talbot, United Kingdom; 277 Department of Urology, Mundakkayam Medical Trust (MMT) Hospital, Mundakkayam, India; 278 Anesthesiology and Critical Care, All India Institute of Medical Science, Bhopal, Madhya Pradesh, India; 279 McGill University Health Centre, Montreal, Québec, Canada; 280 Royal Victorian Eye and Ear Hospital, Melbourne, Austraila; 281 East Surrey Hospital, Surrey and Sussex NHS Foundation Trust, Redhill, United Kingdom; 282 Urology Unit, Department of Surgery, Faculty of Medicine, Universiti Kebangsaan Malaysia (UKM), Kuala Lumpur, Malaysia; 283 Department of Urology, Westmead Hospital, University of Sydney, Sydney, Australia; 284 University of Adelaide, Adelaide, Australia; 285 Sefako Makgatho Health Sciences University, Ga-Rankuwa, South Africa; 286 Klinikum Sindelfingen-Boeblingen, University Medicine Mannheim, Mannheim, Germany; 287 Royal Liverpool Hospital, Liverpool University Hospitals NHS Foundation Trust, Liverpool, United Kingdom; 288 Department of Urology, Allina Health Cancer Institute, Minneapolis, MN, the United States of America; 289 Universiti Technology Mara, Shah Alam, Malaysia; 290 Department of Anesthesiology and Critical Care, Rijnstate Hospital, Arnhem, The Netherlands; 291 Department of Urology, Mayo Clinic, Rochester, MN, the United States of America; 292 3rd Urology Department, National and Kapodistrian University of Athens, “Attiko” University Hospital, Athens, Greece; 293 Urologische Klinik München Planegg, Munich, Germany; 294 Urologische Klinik, Städtisches Klinikum Görlitz, Görlitz, Germany; 295 Department of Urology, Bombay Hospital Institute of Medical Sciecnes, Mumbai, India; 296 Medanta Hospital, Patna, Bihar, India; 297 Department of Urology, Marmara University School of Medicine, Istanbul, Turkey; 298 Department of Urology, Tan Tock Seng Hospital, Singapore; 299 University of Ghana Medical School, Korle Bu Teaching Hospital , Accra, Ghana; 300 Barts Cancer Institute, Queen Mary University of London, London, United Kingdom; 301 Hospital Israelita Albert Einstein, São Paulo, Brazil; 302 Clinica MEDS, Santiago, Chile; 303 University Hospitals Sussex NHS Foundation Trust, Brighton, United Kingdom; 304 Rafina Health Center, Rafina, Greece; 305 Department of Urology, Sapienza University of Rome, Rome, Italy; 306 Lee Kong Chian School of Medicine, Nanyang Technological University, Singapore, Singapore; 307 Yong Loo Lin School of Medicine, National University of Singapore, Singapore, Singapore; 308 Department of Urology, Oxford University Hospitals NHS Foundation Trust, Oxford, United Kingdom; 309 S.H. Ho Urology Centre, Department of Surgery, Prince of Wales Hospital, The Chinese University of Hong Kong, Hong Kong; 310 Department of Urology, Skåne University Hospital, Malmö, Sweden; 311 Urology Department, Royal Surrey County Hospital, Royal Surrey NHS Foundation Trust, Surrey, United Kingdom; 312 Pontificia Universidad Católica de Chile, Santiago, Chile; 313 Hospital Universitary Doctor Peset, Valencia, Spain; 314 University of Texas Southwestern Medical Center, TX, United States of America; 315 Department of Urology, Lausanne University Hospital, Lausanne, Switzerland; 316 S. Chiara Regional Hospital, Locarno, Switzerland; 317 Hirslanden Klinik Beausite, Berne, Switzerland; 318 Department of Surgery, Faculty of Medicine, Khon Kaen University, Khon Kaen, Thailand; 319 Department of Urology, University Hospital Tübingen, Germany; 320 Musgrave Medical, Coolangatta, Australia; 321 Department of Anaesthesiology, Geneva University Hospitals, Geneve, Switzerland; 322 The Royal Wolverhampton NHS Foundation Trust, Wolverhampton, United Kingdom; 323 Klinik für Urologie und Kinderurologie, Katholisches Klinikum Koblenz, Brüderkrankenhaus Montabauer, Montabauer, Germany; 324 Urological Clinic, University Hospital of Padova, Italy; 325 Department of Urology, Academic Hospital Braunschweig, Braunschweig, Germany; 326 Sourasky Medical Center, Tel Aviv University, Tel Aviv, Israel; 327 Department of Urology, Policlinico San Martino Hospital, University of Genova, Genova, Italy; 328 Gaetano Barresi Department of Human and Paediatric Pathology, Urology Section, University of Messina, Messina, Italy; 329 Department of Urology, Städtisches Klinikum Görlitz, Görlitz, Germany; 330 Parkview Hospital, IN, the United States of America; 331 Department of Urology, Emory University, GA, United States of America; 332 University Hospital for Urology, Klinikum Oldenburg, Department of Human Medicine, Carl von Ossietzky University Oldenburg, Oldenburg, Germany; 333 Department of Urology, University of Augsburg, Augsburg, Germany; 334 Ospedale San Bassiano, Bassano del Grappa, Italy; 335 The University Hospital, NHS Ayrshire-Arran, Ayr, United Kingdom; 336 Imelda Hospital, Bonheiden, Belgium; 337 Nepean Hospital, Sydney, Australia; 338 University Medical Center Utrecht, Utrecht, The Netherlands; 339 Department of Urology, Auckland City Hospital, Auckland, New Zealand; 340 Department of Urology, Medical Campus OWL, Universitätsklinikum der Ruhr-Universität Bochum, Herford-Bünde, Germany; 341 IRCCS C.R.O. National Cancer Institute of Aviano, Aviano, Italy; 342 Department of Urology, Instituto Valenciano de Oncologia Fundacion, Valencia, Spain; 343 Department of Surgery, Hospital USM, Universiti Sains Malaysia, Gelugor, Malaysia; 344 University of Ilorin Teaching Hospital, Ilorin, Nigeria; 345 Università Cattolica del Sacro Cuore, Rome, Italy; 346 AdventHealth Global Robotics Institute, Celebration, FL, United States of America; 347 Comprehensive Cancer Center Vienna, Vienna, Austria; 348 Division of Urology, IRCCS Azienda ospedaliero universitaria di Bologna, Bologna, Italy; 349 Urology Department, University Jean Monnet, St. Etienne, France; 350 AU Specialists, Orland Park, IL, the United States of America; 351 Department of Urology, Teikyo University School of Medicine, Tokyo, Japan; 352 Urology Department, Akita University School of Medicine, Akita, Japan; 353 Mater Misericordiae University Hospital, Dublin, Ireland; 354 Fondazione IRCCS Policlinico San Matteo, Pavia, Italy; 355 Royal Derby Hospital, University Hospitals of Derby and Burton NHS Foundation Trust, Derby, United Kingdom; 356 Department of Urology, All India Institute of Medical Sciences, New Delhi, India; 357 School of Medicine, Griffith University, Gold Coast, Australia; 358 University of Eastern Piedmont, Maggiore della Carità Hospital, Novara, Italy; 359 Servicio de Urología, Hospital Universitario Austral, Universidad Austral. Buenos Aires, Argentina; 360 Centro Hospitalar e Universitário de São João, Porto, Portugal; 361 Hôpital Riviera-Chablais (HRC), Rennaz, Switzerland; 362 Division of Urology, Ivrea Civil Hospital (ASL TO4), Ivrea (Turin), Italy; 363 Rahman Medical Institute, Peshawar, Pakistan; 364 Department of Urology, Tauranga Hospital, New Zealand; 365 Hospital Universitario Príncipe de Asturias, Alcalá de Henares, Madrid, Spain; 366 Clínica Alemana, Araucanía, Chile; 367 Urology Department, Freeman Hospital, Newcastle Hospitals NHS Foundation Trust, Newcastle, United Kingdom; 368 Department of Urology, University Hospital Salzburg, Paracelsus Medical University, Salzburg, Austria; 369 National Healthcare Group, Singapore; 370 Muljibhai Patel Urological Hospital, Nadiad, India; 371 Royal Devon and Exeter Hospital Wonford, Royal Devon University Healthcare NHS Foundation Trust, Exeter, United Kingdom; 372 Hospital Santa Casa de São José dos Campos, São Paulo, Brazil; 373 Department of Urology, Institut Mutualiste Montsouris, Paris, France; 374 Faculty of Pharmacy and Medicine Department of Medico-Surgical Sciences and Biotechnologies Urology Unit, ICOT, Sapienza University of Rome, Italy; 375 AO San Giovanni Addolorata, Rome, Italy; 376 Department of Surgical, Oncological and Oral Sciences, Section of Urology, University of Palermo, Palermo, Italy; 377 Urology, Pitie-Salpetriere Hospital, APHP-Sorbonne Université, Paris, France; 378 Department of Urology, University of Patras, Patras, Greece; 379 Department of Oncology, Division of Urology, University of Turin, San Luigi Gonzaga Hospital, Turin, Italy; 380 Division of Urology, Penn Medicine, University of Pennsylvania Health System, Philadelphia, PA, the United States of America; 381 University Hospital Southampton NHS Foundation Trust, Southampton, United Kingdom; 382 Surgical Oncology department, Institut Paoli-Calmettes, Marseille, France; 383 Urology Department, San Giuseppe Hospital, Multimedimedica Group, Milan, Italy; 384 Urology Department, Croix Du Sud Hospital, Quint-Fonsegrive, France; 385 Swedish Medical Group, Seattle, WA, the United States of America; 386 Department of Urology, Stanford University, CA, the United States of America; 387 University of Palermo, Palermo, Italy; 388 Department of Urology, Hospital Universitario Virgen de las Nieves (HUVN), Granada, Spain; 389 Istituto de Investigación Biosanitaria, Granada, Spain; 390 Sindh Institute of Urology and Transplantation, Karachi, Pakistan; 391 Department of Urology, Fondazione Policlinico Universitario Agostino Gemelli, IRCCS, Rome, Italy; 392 Department of Urology, Sir H. N. Reliance Foundation Hospital and Research Centre (HNRFH), Mumbai, India; 393 Department of Urology SLK Kliniken Heilbronn, Heilbronn, Germany; 394 Urology Department, Hospital Universitario Miguel Servet, Zaragoza, Spain; 395 Department of Anaesthesiology, Monash Medical Centre, Melbourne, Australia; 396 Department of Urology, Philipps-University Marburg, Marburg, Germany; 397 Department of Urology, Kantonsspital Aarau, Aarau, Switzerland; 398 Dr. Soetomo General Academic Hospital, Surabaya, Indonesia; 399 Urology Department, Universitario de Galdakao, Galdakao, Spain; 400 Uro-Oncology Unit, Hospital Clinic, University of Barcelona, Barcelona, Spain; 401 Hospital AC Camargo, Cancer Center, São Paulo, Brazil; 402 Department of Urology, Sant Joan de Deu Fundació Althaia Manresa, Spain; 403 Urologische Klinik und Poliklinik, Klinikum der Universität München, Campus Großhadern, Universität München, Munich, Germany; 404 Rochester General Hospital, Rochester, NY, the United States of America; 405 Hospital Central de Lima, Lima, Peru; 406 Klinikum Oldenburg, Carl von Ossietzky University Oldenburg, Oldenburg, Germany; 407 Department of Anesthesia and Intensive Care and Emergency, Città della Salute e della Scienza University Hospital, Turin, Italy; 408 Department of Outcomes Research, Cleveland Clinic, OH, the United States of America; 409 Clinique Pasteur, Toulouse, France; 410 Anesthesiology, Critical Care and Pain Medicine Division, Department of Medicine and Surgery, University of Parma, Italy; 411 Division of Urology, Department of Surgery, McGill University Health Centre, McGill University, Montreal, Canada; 412 Police General Hospital, Bangkok, Thailand; 413 Department of Urology, Northern Health, Broadmeadows, Australia; 414 Prince of Wales Hospital, Sydney, Australia; 415 Department of Urology, Amsterdam UMC, Amsterdam, The Netherlands; 416 Department of Anaesthesiology and Intensive Care Medicine, University Hospital of Cologne, Cologne, Germany; 417 Klinik für Urologie, Uroonkologie, robotergestützte und Fokale Therapie, Universitätsklinikum Magdeburg, University of Magdeburg, Magdeburg, Germany; 418 Servicio de Urología, Centro de Educación Médica e Investigaciones Clínicas “Norberto Quirno” (CEMIC), San Luis, Argentina; 419 Hospital General de Villarrobledo, Albacete, Spain; 420 Klinik für Anästhesie und Intensivmedizin, ev. Krankenhaus Bethesda zu Duisburg, Duisburg, Germany; 421 Department of Urology, Eastern Health Box Hill, Australia; 422 Department of Anaesthesia and Critical Care, Rajiv Gandhi Cancer Institute and Research Centre, Rohini, New Delhi, India; 423 West Hertfordshire Teaching Hospitals NHS Foundation Trust, Watford, United Kingdom; 424 Weill Medical College of Cornell University, New York, NY, United States of America; 425 UT Southwestern, Dallas, TX, the United States of America; 426 Department of Urology, PGIMER, Chandigarh, India; 427 Glasgow Royal Infirmary, NHS Greater Glasgow & Clyde, Glasgow, United Kingdom; 428 Sackler School of Medicine, Tel Aviv University, Tel Aviv, Israel; 429 Deptartment of Urology, Sheba Medical Center, Tel Aviv, Israel; 430 Ospedale S.M.Goretti Asl Latina, Latina, Italy; 431 Fortis Hospital, Mohali, India; 432 Department of Surgical Science, Policlinico Tor Vergata, University of Tor Vergata, Rome, Italy; 433 Charlotte Maxeke Johannesburg Academic Hospital, Johannesburg, South Africa; 434 Klinik für Anästhesiologie und Intensivmedizin, Universitätsklinikum Schleswig-Holstein, Campus Lübeck, Luebeck, Germany; 435 ZorgSaam Hospital, Terneuzen, The Netherlands; 436 St Antonius Ziekenhuis, Nieuwegein, The Netherlands; 437 Newcastle University, Newcastle upon Tyne, United Kingdom; 438 Department of GU oncology, Moffitt Cancer Center, FL, the United States of America; 439 Department of Urology, Hospital Vila Nova Star and Hospital Moriah, Brazil; 440 AZ Groeninge, Kortrijk, Belgium; 441 REGA, Zurich, Switzerland; 442 Pertamina Central Hospital, Jakarta, Indonesia; 443 Department of Urology, Faculty of Medicine, Kafrelsheikh University, Kafr Al Sheikh First, Egypt; 444 Department of Anaesthesiology, National University Hospital (NUH), Singapore; 445 Department of Urology, Hospital Queen Elizabeth, Kota Kinabalu, Malaysia; 446 Ahmadu Bello University Teaching Hospital, Zaria, Kaduna State, Nigeria; 447 Urological Clinic, Department of Medicine, Surgery and Health Sciences, University of Trieste, Trieste, Italy; 448 Division of Anaesthesia, University of Cambridge, Addenbrooke’s Hospital, Cambridge, United Kingdom; 449 Praxis für Urologie Leuggern, Leuggern, Switzerland; 450 Pinderfields General Hospital, Mid Yorks NHS Foundation Trust, Wakefield, United Kingdom; 451 NHS Tayside, Dundee, United Kingdom; 452 Department of Urology, Stadtspital Zurich, Zurich, Switzerland; 453 Johannesburg Academic Urology Centre, Johannesburg, South Africa; 454 Department of Urology, Rijnstate Hospital, Arnhem, The Netherlands; 455 St Antonius Hospital, Utrecht, The Netherlands; 456 Radboud University Medical Center, Nijmegen, The Netherlands; 457 Maxima Medical Centre Veldhoven, Veldhoven, The Netherlands; 458 Policlinico di Bari, Bari, Italy; 459 Princess Alexandra Hospital, Woolloongabba, Australia; 460 Tauranga Urology, Tauranga, New Zealand; 461 Department of Health Sciences, Section of Anaesthesiology, Intensive Care and Pain Medicine, University of Florence, Azienda Ospedaliero Universitaria Careggi, Florence, Italy; 462 Department of Urology and Paediatric Urology, Saarland University, Homburg/Saar, Germany; 463 Department of Anaesthesiology, Intensive Care and Pain Medicine, University Hospital Münster, Münster, Germany; 464 Department of Urology, Rush University Medical Center, Chicago, IL, the United States of America; 465 Clinic for Urology, Pediatric Urology and Andrology, Justus Liebig University Giessen, Giessen, Germany; 466 Nottingham University Hospitals NHS Foundation Trust, Nottingham, United Kingdom; 467 University of Okayama, Okayama, Japan; 468 Division of Urology, Department of Surgery, Royal Thai Navy Hospital, Bangkok, Thailand; 469 Kantonsspital Baselland, Liestal, Switzerland; 470 Kranus Health, Berlin, Germany; 471 Division of Urololgy, University of Cambridge, Addenbrooke’s Hospital, Cambridge, United Kingdom; 472 Australian National University, Canberra, Australia; 473 Bichat-Claude Bernard Hospital, APHP, Paris Cité University, Paris, France; 474 Capital Coast DHB, Wellington, New Zealand; 475 Department of Urology, Biella Hospital, Biella, Italy; 476 Department of Surgical, Oncological and Gastroenterological Sciences, Urological Clinic, University of Padua, Padua, Italy; 477 Hospital Regional Louis Pasteur, Villa María, Argentina; 478 Fujita Health University, Tokyo, Japan; a Department of Urology, Royal Melbourne Hospital, University of Melbourne, Parkville, Australia; b Department of Urology, Western Health, Melbourne, Australia; c Department of Anaesthesiology and Pain Medicine, Inselspital, Bern University Hospital, University of Bern, Bern, Switzerland; d Department of Urology, Solothurner Spitäler AG, Olten/Solothurn, Switzerland; e Department of Urology, Inselspital, Bern University Hospital, University of Bern, Bern, Switzerland

**Keywords:** Complication reporting, Urological surgery, Delphi method, Surgical difficulty, Risk estimation

## Abstract

Surgical complications remain a major source of preventable morbidity, mortality, and health care expenditure, but existing frameworks such as the Clavien-Dindo classification and Comprehensive Complication Index are clinician-centred and intervention-focused and fail to capture cumulative patient-centred outcomes. This protocol outlines the Complications After Major and Minor Urological Surgery (CAMUS) initiative, a global, multiphase effort to redefine complication reporting, risk stratification, and outcome measurement in urological surgery. CAMUS aims to address these limitations via an integrated, seven-arm programme combining retrospective and prospective data analysis, consensus development, and digital infrastructure design. Arm 1 has assembled a retrospective data set of 130 034 major urological procedures from 180 centres across 33 countries, the largest of its kind. Arms 2 and 3 have completed Delphi surveys with physicians (*n* = 1113) and pilot nursing participants (*n* = 20) and has generated consensus on novel grading domains and highlighted the importance of multidisciplinary perspectives. Arm 4 will incorporate patient-reported outcomes and behavioural economics methods to quantify subjective burdens, while arm 5 will develop the CAMUS Intraoperative and Postoperative Risk and Difficulty Estimation Index (IPRADES) for surgical risk and difficulty prediction. Arms 6 and 7 will build a secure e-database and dictionary and prospectively validate the system using >2000 new cases. Statistical methods include multivariable regression, meta-analysis of individual patient data, and machine-learning approaches to model predictors of morbidity and mortality. Outputs will be benchmarked internationally to facilitate both clinician- and patient-driven definitions of complication severity. Ultimately, CAMUS will deliver a reproducible, patient-inclusive classification system with broad applicability to clinical practice, audits, education, and policy. By integrating more than 130 000 procedures with global Delphi consensus, CAMUS represents the most comprehensive complication classification initiative undertaken in surgery. Its outputs are expected to improve transparency, standardise reporting, and inform patient-centred risk stratification worldwide.

## Take Home Message

The CAMUS initiative has established the first global, multiphase, and multidisciplinary framework to redesign how surgical complications in urology are defined, graded, and reported. By integrating surgeon, nursing, and patient perspectives, harmonising international data sets, and incorporating novel concepts such as sequential, cumulative, and intraoperative complications as well as grading of surgical difficulty, CAMUS aims to deliver a validated and universally applicable classification system. This protocol outlines the roadmap towards a more transparent, patient-centred, and clinically meaningful standard for reporting complications in urological surgery worldwide.

## 1 Introduction and hypotheses

Surgical complications remain a leading cause of preventable morbidity, mortality, greater health care costs, and lower patient satisfaction [1], [2]. As the global volume of surgical procedures continues to rise [3], the consequences of complications for both patients and health care systems are becoming increasingly prominent. Complications can result in prolonged hospital stays, unplanned readmissions, additional interventions, long-term disability, and even death. From a health-care system perspective, these events increase costs and place additional pressure on resources. For clinicians, they represent missed opportunities to deliver safe, high-quality care; for patients, they may significantly reduce trust in surgical services and contribute to physical and emotional distress.

Despite advances in surgical techniques, anaesthesia, and perioperative medicine, a critical issue persists: the lack of a universally accepted, consistent, and patient-inclusive system for classifying and reporting surgical complications. Accurate and reproducible classification is not only essential for benchmarking and quality improvement but also fundamental to effective patient counselling and informed consent. The absence of a standardised, comprehensive complication classification system limits our ability to compare outcomes across centres, identify areas for improvement, and ensure transparency in academic reporting and clinical decision-making [4], [5], [6], [7].

While systematic registration of complications may initially be perceived as burdensome, much of the current workload arises from fragmented, duplicative documentation across multiple parallel systems. CAMUS aims to consolidate complication reporting into a single structured framework that will allow one high-quality record to serve clinical documentation, audits, education, and research simultaneously. Experience from early adopters suggests that after an initial learning phase, this approach reduces the overall workload rather than increasing it.

Standardised complication registration also plays a critical role in surgical training and education. Transparent documentation of perioperative morbidity exposes trainees to the full spectrum of surgical risk, including minor, cumulative, and delayed complications that are often under-represented in traditional operative logs. Structured complication data support reflective practice, objective assessment of learning curves, and competency-based progression, while also informing appropriate case allocation and supervision within training programmes.

The Clavien-Dindo classification (CDC) is currently the surgical complication grading system most widely used [8]. The CDC categorises complications according to the type of therapeutic intervention required, which ranges from minor deviations from the normal postoperative course (grade I) to death (grade V). The Comprehensive Complication Index (CCI) sought to improve on this framework by aggregating all complications experienced by a patient into a single weighted score. However, both systems are fundamentally clinician-centred and intervention-focused, and frequently neglect complications that do not require active medical or surgical treatment such as fatigue, chronic pain, and sexual dysfunction. Furthermore, intraoperative complications are not captured by the CDC.

In addition, these systems do not adequately account for procedure-specific contexts or the clinical relevance of complications relative to the operation performed. A given intervention may have markedly different implications depending on the procedure, anatomic site, and patient expectations. This limitation was a primary motivation for the CAMUS initiative, which was designed to capture event-level detail, cumulative morbidity, and procedure-specific nuance and thus allow more clinically meaningful interpretation and comparison of outcomes across diverse urological surgeries.

Moreover, the CDC and CCI only allow documentation of the highest-grade complication per patient per surgical episode, and thus obscure the cumulative burden of multiple lesser complications. This has significant implications for patient outcomes and recovery experiences, and the accuracy of institutional and interventional benchmarking. In addition, these systems do not integrate patient-reported outcome measures (PROMs) or patient experience metrics and often fail to capture complications occurring beyond 30 d or 90 d postoperatively [9], [10], which are particularly relevant for high-morbidity procedures such as radical cystectomy [5]. The under-representation of late, recurrent, or subjective complications poses challenges for both clinical research and real-world patient care.

Multiple systematic reviews of the literature have identified wide heterogeneity in complication reporting in urology. For example, 30-d and 90-d complication rates following radical cystectomy vary significantly between studies and range from 26% to 86%, and from 30% to 100%, respectively [11], [12], [13], [14], [15], [16], [17]. While differences in surgical complexity, patient comorbidity, and institutional expertise partly explain this variation, it is widely assumed that inconsistency in complication documentation and classification plays a greater role. No current system has reached universal acceptance in the urology community, and few have undergone large-scale prospective validation. Even among validated frameworks such as the CDC, ambiguity remains in how to define, grade, and report complications that are serial or recurrent or require multiple interventions to resolve [18], [19], [20].

A key challenge in developing any complication reporting system is balancing sufficient clinical detail with usability and feasibility. Excessive complexity risks poor adoption and incomplete reporting, whereas oversimplification obscures clinically meaningful information. CAMUS addresses this by adopting a modular design, with a simple core data set suitable for routine clinical use and optional advanced layers that facilitate granular documentation when required for research, benchmarking, or education.

In addition, there is no consensus on how to report so-called “complication-intervention events”, which are specific combinations of a clinical complication and the therapeutic action taken in response. While these events are highly relevant to patients and clinicians alike, their reporting remains variable and often poorly defined in the literature. Furthermore, there is growing recognition that traditional classification frameworks overlook the perspectives of nursing staff and patients, both of whom provide critical insight into early complication signs, the subjective burden, and the impact on quality of life (QoL).

To address these longstanding challenges, the CAMUS project (Complications After Major and Minor Urological Surgery) was launched in 2019 by a global, multidisciplinary consortium of urologists, anaesthetists, nurses, behavioural scientists, and patient advocates [21]. The aim of CAMUS is to develop a comprehensive, standardised, and patient-inclusive system for classifying surgical complications [21], [22]. This initiative builds on existing evidence and practice but seeks to fill critical gaps via a series of interlinked research arms that include stakeholder consensus development, real-world data analysis, the creation of digital infrastructure, and prospective validation [9], [23].

The CAMUS initiative was established via targeted competitive research funding obtained during dedicated research fellowships to support methodological development, consensus processes, and infrastructure design. Since inception, CAMUS has been sustained via investigator-driven academic collaboration to ensure scientific independence. Ongoing and future sustainability will be supported via additional competitive funding, shared institutional ownership, and integration into routine audit and digital workflows.

CAMUS was designed as a multiarm study incorporating both retrospective and prospective data collection, Delphi consensus studies with physicians and nurses, behavioural economics–informed patient surveys, and the development of a novel surgical difficulty and risk index. With contributions from 180 centres across 33 countries, CAMUS encompasses data for more than 130 000 major urological procedures [21], [22], [24], [25], [26], [27], [28]. This unprecedented international scope makes it the largest complication classification development project in surgery to date and ensures that the framework is grounded in real-world diversity of practice and patient populations.

Although initially developed within urology, CAMUS was conceived from the outset as a specialty-agnostic framework. Its principles of comprehensive event capture, intervention-linked grading, and patient-inclusive reporting are applicable across surgical disciplines. Future phases of CAMUS are explicitly designed to support extension to other procedural specialties, particularly those involving major abdominal and pelvic surgery, to facilitate cross-specialty benchmarking and broader adoption of a unified complication reporting language.

The goals of CAMUS are fourfold:

1. To create a conclusive and reproducible language for complication reporting via international Delphi consensus that incorporates expert opinions from surgeons, anaesthetists, perioperative nurses, and patients themselves;
2. To build and maintain a large multinational database of surgical complications that allows for robust statistical analysis, prediction modelling, and interinstitutional benchmarking;
3. To develop validated intraoperative and perioperative risk and difficulty indices for more accurate surgical planning, patient counselling, and resource allocation; and
4. To prospectively validate the CAMUS classification system and associated digital tools that integrate PROMs and other patient-centred metrics to ensure that reporting reflects real-world complication burdens.

In addition to improving classification, the development of accurate risk prediction models may help to explain variation in complication rates by incorporating differences in patient comorbidity, surgical difficulty, and institutional characteristics [29]. Effective risk stratification allows preoperative identification of high-risk cases and facilitates multidisciplinary decision-making, including the allocation of intensive care resources, surgical planning, and tailored postoperative care pathways [30], [31]. It also enhances intersurgeon and interinstitutional comparisons, which are vital for quality benchmarking and performance review [32]. Moreover, the integration of PROMs and patient perspectives into complication reporting ensures a more holistic evaluation of outcomes that reflects not only clinical events but also functional and QoL consequences that matter most to patients [33].

## 2 Study design and framework

**Fig. 1:**
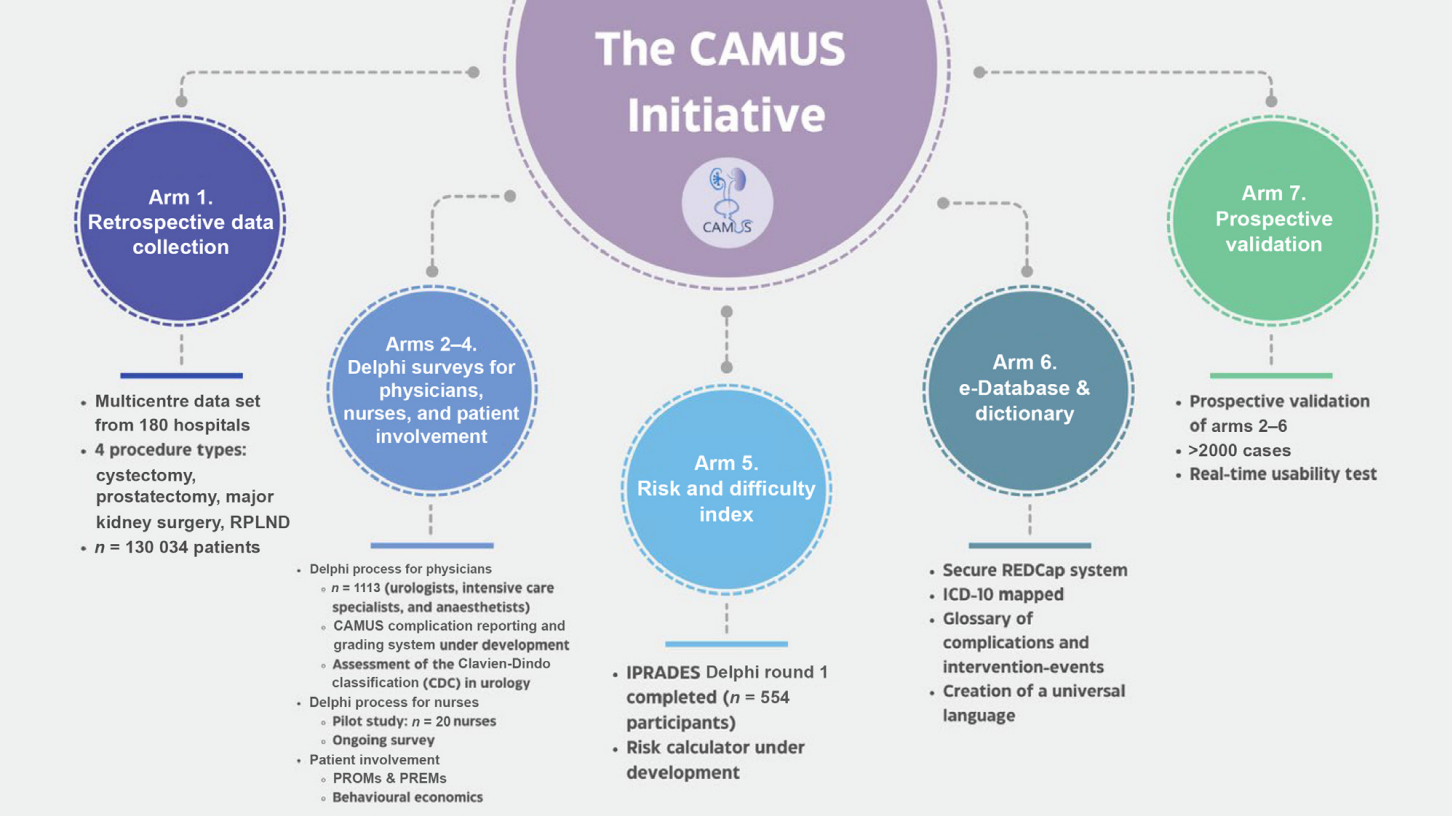
Overview of the seven research arms in the CAMUS Initiative, illustrating the multiphase, international framework for complication reporting, consensus development, risk stratification, database creation, and prospective validation. ICD = International Classification of Diseases; IPRADES = Intraoperative and Postoperative Risk and Difficulty Estimation Index; PREMs = patient-reported experience measures; PROMs = patient-reported outcome measures; REDcap = Research Electronic Data capture; RPLND = retroperitoneal lymph node dissection.

The CAMUS initiative is structured as a multiphase, multicentre international cohort study that includes retrospective and prospective collection of data, the development of stakeholder consensus, the design of digital infrastructure, and validation analysis. The modular design comprises seven coordinated yet distinct study arms, each of which contributes to the overall development and implementation of the CAMUS classification system (Table 1 and Fig. 1).

**Table 1:** Overview of the seven CAMUS study arms: design and progress to date

Arm	Title	Objective	Methods	Status
Arm 1	Retrospective data collection	Collect real-world data on major urological procedures and postoperative outcomes	Retrospective chart review from 180 hospitals; 130 034 cases across 4 procedure types	Data collection complete; analyses and validation ongoing
Arm 2	Delphi study: physicians	Achieve international expert consensus on complication classification and grading	REDCap-based Delphi survey with 1113 clinicians from 57 countries, ≥75% consensus	Initial Delphi study complete; framework refinement and validation planned
Arm 3	Delphi study: nursing staff	Integrate nursing perspectives into complication reporting and classification	Pilot Delphi round with 20 nurses assessing feasibility and thematic insights	Pilot complete; large-scale Delphi planned
Arm 4	Patient involvement	Assess patient-perceived burden and value of complications using PROMs and economic tools	Digital survey of PROMs, VAS, and WTP tools to measure subjective complication burden	Planned
Arm 5	Risk and difficulty index	Develop a risk and difficulty index for surgical planning based on expert Delphi consensus	Structured Delphi consensus to weight and validate the IPRADES scoring model	Preliminary Delphi study complete; further Delphi study and validation planned
Arm 6	e-Database and Dictionary	Design a digital data capture system and dictionary aligned with ICD and CAMUS grading	Database fields mapped to ICD codes; structured data entry for real-time analysis	Planned
Arm 7	Prospective validation	Pilot real-time implementation and comparison of CAMUS to traditional grading systems	Real-time entry of >2000 cases; feedback on usability, sensitivity, and acceptability	Planned

ICD = International Classification of Diseases; IPRADES = Intraoperative and Postoperative Risk and Difficulty Estimation Index; PROMs = patient reported outcome measures; REDcap = Research Electronic Data capture; VAS = Visual Analogue Scale; WTP = willingness to pay.

### 2.1 Arm 1: retrospective data collection

To date, discrete data sets have been collated for 130 034 patients who have undergone major urological procedures (radical cystectomy with urinary diversion, radical prostatectomy, major kidney surgery, and retroperitoneal lymph node dissection [RPLND]) at participating centres worldwide. These data include patient demographics, comorbidities, surgical variables, and postoperative outcomes, with complications graded according to the CDC.

By design, endoscopic procedures such as transurethral resection of bladder tumour and transurethral resection of the prostate were deliberately excluded from the initial validation phase to allow testing of the CAMUS framework in high-complexity, high-morbidity surgeries for which complication-intervention pathways are more heterogeneous. Inclusion of lower-risk endoscopic procedures is planned in subsequent phases once the system has been fully validated in major surgery.

Moving forward, the objectives of this arm are as follows:

o To generate robust, clinically relevant insights into postoperative outcomes by analysing the international data set;
o To externally validate established complication scoring systems, including the CDC and CCI, and assess their consistency, sensitivity, and applicability across diverse health care settings;
o To conduct patient-level meta-analyses and multivariable regression to identify independent predictors of morbidity as a foundation for predictive models and a validated risk index for preoperative assessment;
o To facilitate anonymous benchmarking across hospitals, health care systems, and regions worldwide, with identification of structural and procedural factors driving variability in complication rates; and
o To use the large-scale data set to address a range of clinically relevant research questions, with the ultimate aim of improving surgical risk stratification, outcome prediction, and perioperative care worldwide.

### 2.2 Arm 2: Delphi study for physicians

A structured international Delphi survey was conducted in 2022 that involved 1113 clinicians from 57 countries, with consensus achieved on key domains for complication reporting and grading. The survey was performed under the guidance of the CAMUS Steering Committee. Participant experts were contacted via e-mail (with addresses obtained via CAMUS databases and trial coordinators) and word of mouth. Consensus was predefined as ≥75% agreement across rounds.

Moving forward, the Delphi outputs will guide the establishment of a new CAMUS complication grading and reporting system. This will involve refinement and expansion of the CDC, with integration of the new CAMUS domains and supplemental grades, and the creation of a unified framework tailored for urological surgery. The revised system will then be validated against the full CAMUS registry of >130 000 patients, which will allow direct comparison of CAMUS and CDC performance across procedures, centres, and health care systems [24]. This will provide evidence for the sensitivity, reproducibility, and international applicability of the CAMUS framework.

### 2.3 Arm 3: Delphi study for nursing staff

A dedicated Delphi survey with specialist urology nurses was conducted in 2022 to capture their perspectives on complication reporting and grading. The pilot phase, performed under guidance by the CAMUS Steering Committee, included 20 participants and revealed strong support (75%) for the CAMUS system and universal recognition of the need for reporting consensus.

Moving forward, the nursing Delphi survey will be expanded to several hundred participants across multiple countries and practice settings. This broader cohort will capture diverse perspectives from perioperative, ward-based, and advanced practice nurses in urology. The goal is to embed nursing expertise into the CAMUS framework by defining how nurses identify, grade, and document complication-intervention events. This will ensure that bedside recognition of morbidity, minor but clinically significant interventions, and QoL-related outcomes are appropriately represented. The expanded survey will also assess training needs, the feasibility of integration into nursing workflows, and barriers to adoption. Findings will directly inform the final CAMUS reporting and grading system, which will thus be inclusive of all professional groups involved in perioperative care [28].

### 2.4 Arm 4: patient involvement

Patients undergoing urological surgery will be surveyed to capture PROMs and patient-reported experience measures). Validated tools such as the 36-item Short Form survey on general health (SF-36), International Index of Erectile Function-15 (IIEF), Female Sexual Function Index (FSFI), International Consultation on Incontinence Questionnaire (ICIQ), and Gastrointestinal Quality of Life Index (GIQLI) will be used to assess QoL domains. These will be complemented by behavioural-economics instruments, including willingness to pay (WTP) and perceived value frameworks, which will quantify the subjective burden of complications and identify which outcomes matter most to patients.

Data will be analysed to construct a Patient-Centred Burden Index, which will integrate with clinician-derived CAMUS grading and ensure that classification reflects both objective morbidity and patient-valued outcomes.

### 2.5 Arm 5: surgical risk and difficulty index

A Delphi survey process, guided by the CAMUS Steering Committee and involving surgeons, anaesthetists, and methodologists, was conducted in 2025 to develop the CAMUS Intraoperative and Postoperative Risk and Difficulty Estimation Index (IPRADES) tool, which is a comprehensive scoring system for predicting surgical difficulty and perioperative risk in urology. The index incorporates preoperative, intraoperative, and immediate postoperative factors and takes into account for organ-specific variables, surgeon experience, and surgical complexity [26].

Moving forward, the IPRADES Delphi process will be expanded to several hundred international participants to achieve broad consensus and ensure applicability across diverse practice environments. Responses will be analysed to refine item weighting, with multivariable regression modelling used to validate discriminatory accuracy against outcomes such as high-grade complications, intensive care unit (ICU) admission, transfusion, and length of stay. Once calibrated using the CAMUS data set, the final IPRADES score will be categorised into low-risk, moderate-risk, and high-risk strata. It will be implemented as a web-based calculator to guide case selection, patient counselling, benchmarking of surgical difficulty, and allocation of perioperative resources [27].

### 2.6 Arm 6: CAMUS e-database and dictionary

A secure, user-friendly electronic database will be built to standardise complication reporting across centres. This database will be structured around a CAMUS data dictionary that will incorporating both urology-specific and general complications mapped to International Classification of Diseases v10 (ICD-10) codes where possible. The database will allow time-stamped entry of multiple complications per patient, will link complications to interventions, and will accommodate supplemental grades and PROMs.

Automated export to statistical software and real-time dashboards will support clinical audits, benchmarking, and research. The platform will also include audit trails, role-based access, and encryption to ensure compliance with international data governance standards.

### 2.7 Arm 7: prospective validation

The CAMUS classification and database will undergo large-scale prospective validation following pilot testing. Participating centres will enter data in real time for >2000 new surgical patients for assessment of usability, inter-rater reliability, and completeness of reporting. Outcomes from the CAMUS system will be compared directly to the CDC and CCI to evaluate sensitivity, reproducibility, and alignment with PROMs.

Stakeholder feedback will guide iterative refinement of the dictionary and interface to ensure the feasibility of integration into routine surgical audits and education. This phase is intended to confirm that CAMUS is scalable, reliable, and internationally applicable.

Each arm was independently designed and conducted, with harmonisation overseen by a global steering committee. While the CAMUS methodology is comprehensive, its modular design allows centres to adopt the framework at a level appropriate to their resources. A simplified core data set supports routine clinical use, with advanced components activated selectively. This scalability ensures applicability across diverse health care environments while preserving methodological rigour.

## 3 Protocol overview: data collection and management

### 3.1 Retrospective arm (arm 1)

The CAMUS initiative involved 180 hospitals across 136 cities in 33 countries spanning six continents. The largest number of contributing centres was in Europe (*n* = 96), followed by North America (*n* = 23), Oceania (*n* = 23), Asia (*n* = 21), South America (*n* = 12), and Africa (*n* = 5). This mix of academic, private, and regional centres provides a diverse representation of surgical practice, infrastructure, and patient populations (Table 2). Adult patients (aged ≥18 yr) undergoing major urological surgeries were included via both retrospective chart review and prospective enrolment.

**Table 2:** Geographic distribution of centres contributing to the CAMUS multicentre database by continent and country

Parameter	Centres (*n*)
Centres contributing data by continent	
Europe	96
North America	23
Oceania	23
Asia	21
South America	12
Africa	5
Centres contributing data by country	
Germany	28
Australia	22
UK	18
US	18
Italy	13
France	9
Brazil	8
Switzerland	7
India	6
Canada	5
Belgium	6
Spain	4
Netherlands	4
South Africa	3
Japan	3
Argentina	3
Republic of Singapore	3
Turkey	3
Czechia	2
Austria	2
Taiwan	1
New Zealand	1
China	1
Ghana	1
Chile	1
Indonesia	1
Greece	1
Slovakia	1
Malaysia	1
Israel	1
Sweden	1
Egypt	1
Thailand	1
Number of cities/towns	136
Number of hospitals	180

In total, 130 034 major urological procedures were captured (Table 3):

• Radical cystectomy with urinary diversion: 29 098 cases from 126 centres.
• Radical prostatectomy: 75 001 cases from 111 centres.
• Major kidney surgery (partial nephrectomy, radical nephrectomy, nephroureterectomy): 24 476 cases from 107 centres.
• RPLND: 1459 cases from 30 centres.

**Table 3:** Baseline procedural data for 130 034 patients in the CAMUS multicentre database: number of contributing hospitals and patient numbers for cystectomy, prostatectomy, major kidney surgery, and retroperitoneal lymph node dissection

Procedure	Number
Cystectomy	
Hospitals contributing cystectomy data	126
Patients who underwent cystectomy	29 098
Prostatectomy	
Hospitals contributing prostatectomy data	111
Patients who underwent prostatectomy	75 001
Major kidney surgery	
Hospitals contributing major kidney surgery data	107
Patients who underwent major kidney surgery	24 476
Radical nephrectomy	8832
Partial nephrectomy	13 670
Nephroureterectomy	1974
RPLND	
Hospitals contributing retroperitoneal lymph node dissection data	30
Patients who underwent retroperitoneal lymph node dissection	1459

These procedures, performed between 1978 and 2025, represent the largest international data set of its kind in urological surgery. This will provide a uniquely rich platform for benchmarking outcomes across healthcare systems and inform the development and validation of the CAMUS classification.

Participating sites represented diverse geographic and institutional settings, including academic centres, private hospitals, and regional surgical units. Exclusion criteria were patients aged <18 yr, those unable to provide informed consent, and individuals with unreliable data because of advanced illness or cognitive impairment.

Data elements captured patient demographics, comorbidities, operative characteristics, and postoperative outcomes, with complications initially graded using the CDC, CCI, and Bern CCI before being mapped to the CAMUS system. Surgeon experience and centre-level procedural volume metrics were also captured in anonymised form for analysis of volume-outcome relationships and training-stage effects in later CAMUS study arms.

Data were collected for the following variables:

• Preoperative: age, sex, body mass index, American Society of Anesthesiologists (ASA) score, Charlson Comorbidity Index, diabetes status, smoking status, renal function, anaemia, prior abdominal surgery, and perioperative therapies (eg, neoadjuvant chemotherapy or radiotherapy).
• Intraoperative: procedure type, surgical approach (open, laparoscopic, robotic), operative time, estimated blood loss, transfusion requirements, and intraoperative complications.
• Postoperative: pathology, nodal staging, hospital length of stay, readmissions, death, complication type and timing, intervention, and CDC grade.

Non-identifiable data were encrypted and stored on secure servers at the Royal Melbourne Hospital in compliance with international data governance standards. Data were managed in Research Electronic Data capture (REDCap), which allows structured field entry with logic checks, dropdown menus, and enforced definitions to minimise errors. Each record was time stamped and version-controlled with a full audit trail. Batch import functionality allowed large-scale institutional uploads, while custom scripts supported export to R, SPSS, and Stata for downstream analysis [34], [35].

Future directions for arm 1 include external validation of existing classification systems (CDC, CCI) and the CAMUS classification system across diverse populations, identifying predictors of morbidity through patient-level meta-analysis and regression modelling, and enabling benchmarking across hospitals and continents, revealing structural and procedural factors associated with variation in outcomes. These analyses will provide the empirical foundation for the CAMUS framework, inform the development of predictive indices, and highlight opportunities to improve perioperative outcomes globally.

Participation in CAMUS is voluntary, and early adopters may be more likely to be academically engaged or quality-focused, which may introduce potential selection bias. However, this is a pragmatic and necessary feature of early framework development to ensure data quality and feasibility. The inclusion of centres from 33 countries across diverse health care systems mitigates, but does not eliminate, this effect. As automation and integration improve, broader participation is anticipated.

### 3.2 Delphi survey for physicians (arm 2)

Between 2020 and 2023, a multiround Delphi survey was conducted to establish international consensus on complication classification and reporting, with the specific aim of refining the CAMUS system. A total of 1113 clinicians from 57 countries, including consultant urologists, trainees, anaesthetists, and intensive care specialists, participated. Surveys were administered via REDCap, structured into 12 modules with Likert scales, multiple-choice questions, and free-text responses. Consensus was predefined as agreement of ≥75%.

Round 1 achieved broad engagement. Participants expressed strong agreement on the need for serial and multistage complication tracking, integration of intraoperative and extended grading, minor and long-term outcomes, disability-adjusted severity modifiers, and patient-reported metrics. Free-text responses were coded independently, synthesised, and ranked before being iteratively presented again in later rounds to achieve convergence.

To build on these findings, CAMUS will formalise a new complication grading and reporting system by refining and extending the CDC via supplemental CAMUS domains (intraoperative, postoperative, extended, and disability-adjunct grades). In parallel, a 0–100 continuous scale will be developed to underpin the CAMUS CCI to allow quantification of cumulative morbidity at the individual patient level.

The revised framework will be validated against the >130 000-patient CAMUS registry to facilitate comparative analyses with the CDC and CCI across procedures, institutions, and continents. These evaluations will test the reproducibility, inter-rater reliability, and sensitivity to patient-centred outcomes. Ultimately, outputs will be synthesised into consensus-driven recommendations and published as international guidelines to provide a foundation for standardised surgical audits, research, and perioperative counselling worldwide.

### 3.3 Delphi survey for nursing staff (arm 3)

A pilot Delphi round was completed with 20 experienced inpatient and outpatient nurses specialised in urology, perioperative nurses, and urology-specific advanced practice nurses/nurse practitioners. The survey addressed complication recognition, intervention documentation, training needs, and the feasibility of structured nurse participation. The results demonstrated universal support for the development of a standardised classification system. All participants recognised its necessity, and 75% agreed on adoption of the proposed CAMUS framework. Nurses emphasised the importance of their inclusion in classification development, particularly for early bedside detection of morbidity, documentation of interventions that may not require physician input, and management of complications with significant QoL impact such as wound issues, stoma care, and continence. Barriers were also identified, including inconsistent training, under-recognition of nurse-relevant complications, and the lack of structured reporting tools.

To build on these insights, the nursing Delphi process will now be expanded to several hundred participants across multiple continents. Recruitment will deliberately encompass perioperative nurses, ward-based staff, nurse practitioners, and advanced practice nurses to ensure broad representation of roles and practice settings. The survey will test refined definitions, complication-intervention pairings, and reporting tools tailored to the unique contributions of nursing staff to the recognition and management of complications. Specific domains will include early identification of clinical deterioration, documentation practices, the feasibility of integration into daily workflows, and assessment of training needs and barriers to adoption. Findings will be embedded into the CAMUS dictionary and grading schema to ensure that nursing expertise is incorporated into the final framework. This will guarantee that arm 3 of CAMUS reflects the multidisciplinary reality of perioperative care and captures a more complete picture of patient morbidity.

### 3.4 Patient input (arm 4)

Patients from CAMUS collaborative centres will be invited to participate via digital survey platforms. Eligible participants will include adults (aged ≥18 yr) who have undergone major urological surgery within the previous 12 mo. To capture health-related QoL, validated PROMs will be used, including the SF-36 for general health, IIEF-15, FSFI, ICIQ for urinary continence, and GIQLI for gastrointestinal QoL.

These validated tools will be supplemented by custom-designed behavioural economics modules, such as WTP to avoid specific complications and reverse-WTP or refund preference models related to adverse outcomes. Patients will also complete Visual Analogue Scale (VAS) questionnaires to rate the perceived burden of complications and participate in scenario-based assessments (eg, upgrade and refund simulations) to quantify subjective outcome preferences and complication severity. For instance, postprostatectomy patients may be asked how much they would pay for guaranteed restoration of continence or erectile function, or what refund they would expect if long-term complications occurred.

Findings will be synthesised into a patient-centred subjective burden index designed to complement clinician-derived grading within the CAMUS classification. By incorporating patient valuations, this index will help in redefining what constitutes a “major” or “bothersome” complication, particularly in cases that providers might otherwise categorise as “minor”.

### 3.5 Development of a risk and difficulty index (arm 5)

Underpinned by the published protocol paper, IPRADES is being developed as a CAMUS organ- and procedure-specific tool to anticipate surgical difficulty and estimate perioperative risk in major urological operations. Its purpose is to facilitate standardised assessment of case complexity, training value, and perioperative triage.

Phase 1 was completed using a structured Delphi methodology with 60 high-volume urologists, anaesthetists, and perioperative methodologists. Participants rated 40 candidate variables across four domains: (1) preoperative factors, such as tumour size and stage, organ-specific anatomic complexity (eg, RENAL nephrometry score, prostate volume), prior surgery or radiotherapy, neoadjuvant treatment, comorbidities (ASA score, Charlson Comorbidity Index, body mass index); (2) general intraoperative complexity, including surgical approach (open vs minimally invasive), adhesions or fibrosis, anatomic variants, blood loss, transfusion, operative duration, and local invasion; (3) organ-specific intraoperative findings; and (4) immediate postoperative course indicators, such as ICU admission, early reintervention within 72 h, transfusion, and early complications (optional extension, not required for prospective predictive modelling). Each parameter was rated for clinical relevance and procedural impact using a 10-point Likert scale, with ≥75% agreement required for consensus. Median and interquartile range statistics were used to test stability, with Kruskal-Wallis subgroup analyses performed by discipline and region. Delphi outputs confirmed alignment between surgical and anaesthetic perspectives.

In phase 2, retrospective multicentre data sets will be analysed: >1500 cystectomy cases from the Bern database first, followed by validation in the >130 000-patient CAMUS data set. Associations with predefined outcomes (CDC grade ≥III, CCI ≥33.7, ICU admission) will be examined to define thresholds and calibrate risk weightings.

Phase 3 will involve multivariate logistic regression modelling, with predictive performance assessed via receiver operating characteristic (ROC) curves, calibration plots, c-index metrics, and analysis of the area under the ROC curve (AUC). Stepwise model selection will be based on minimisation of the Akaike information criterion (AIC), while bootstrapping and k-fold cross-validation will mitigate overfitting and test robustness. The final model will be visualised using nomograms (*rms* package in R) and generate a cumulative difficulty score (scale 0–100) stratified into low, moderate, and high tiers, with adjustments for surgeon experience.

Pilot testing will assess construct validity and feasibility, followed by prospective validation across CAMUS centres. Anticipated applications include preoperative communication of risk to patients, surgical planning, benchmarking of procedural difficulty, triaging of teaching cases, and forecasting of resource allocation for ICU and high-dependency units.

### 3.6 e-Database and dictionary (arm 6)

The CAMUS e-database will be implemented as a secure, web-based platform using REDCap infrastructure with structured and encrypted data-entry fields. A dynamic data dictionary mapped to ICD-10 codes and urological terminology will guide standardised entry of complications, interventions, and PROMs. The dictionary will encompass both common urological complications (eg, anastomotic leak, haematuria, infection) and general postoperative issues (eg, pneumonia, myocardial infarction).

Governance of the CAMUS dictionary will be overseen by the CAMUS Steering Committee. New complication-intervention events submitted by participating centres will undergo expert review before integration to ensure version control, consistency, and transparency. This structure enables arm 6 to function as a living, continuously updated resource.

The system will support entry of multiple complications per patient, with automatic time-stamping of events and linkage to interventions. Severity will be graded in parallel using CDC, CCI, Bern CCI, and the new CAMUS scale. Complication timelines, readmissions, and patient-reported burden data will be mapped for integrated analysis.

Planned features include a dynamic interface for real-time auditing, automated export to statistical software, and interactive dashboards for morbidity and mortality review, benchmarking, and educational purposes. The e-database will undergo pilot testing to evaluate usability, data integrity, and workflow integration, with iterative refinement based on feedback from end-users and participating institutions.

### 3.7 Prospective validation (arm 7)

In future implementation phases, the CAMUS classification and digital infrastructure will be prospectively applied across participating centres. The project aims to capture data from more than 2000 new patients entered in real time for formal validation of the system against traditional classification frameworks. Validation will assess inter-rater reliability, predictive accuracy, alignment with PROMs, and the ability to capture nuanced, multistage complications.

Centres will be invited to provide structured feedback on usability, the data burden, and integration into routine surgical audit workflows. It is anticipated that CAMUS will offer better resolution of complication tracking, greater granularity, enhanced recognition of complications often under-reported in legacy systems, and high acceptability among both clinicians and patients.

### 3.8 Ethics and regulatory approvals

Recruitment of centres to process institutional data bases and collect data to characterise complication incidence was undertaken between August 2019 and February 2025. Ethics approval was granted by Melbourne Health (QA2020046) and Epworth HealthCare (EH2021-708). The study was registered on ClinicalTrials.gov (NCT04976946) on August 12, 2021. Additional methodological support was sought from relevant international bodies, including the European Association of Urology.

## 4 Statistical analysis

### 4.1 Statistical inference and risk factor screening

To identify independent risk factors for postoperative complications, statistical analyses will be selected according to the data distribution and study design. Continuous variables will be reported as the median and interquartile range, and compared using nonparametric tests (eg, Mann-Whitney U or Kruskal-Wallis test) unless normality assumptions are met. Categorical data will be expressed as the frequency and percentage, and compared using χ^2^ or Fisher’s exact tests, as appropriate. Correlations will be assessed using Spearman’s rank coefficient. Where relevant, multiple comparisons will be adjusted using standard correction methods (eg, Bonferroni or false discovery rate).

### 4.2 Systematic meta-analysis of individual patient data

In the first study arm (retrospective cohort data) we will use a meta-analysis of individual patient data (IPD) to investigate clinical outcomes and predictive factors across a large cohort of patients undergoing urological procedures. By pooling raw, patient-level data from multiple studies and centres, we aim to overcome limitations inherent to traditional meta-analyses of aggregate data, such as inconsistent variable definitions and lack of adjustment for individual-level covariates.

Our objectives are to (1) derive more precise estimates of key outcomes, (2) identify and validate predictors of perioperative and long-term complications, and (3) explore potential interactions or subgroup effects that could inform personalised risk stratification and clinical decision-making. This comprehensive analysis will use the strengths of IPD to provide robust, generalisable evidence that could improve patient counselling, risk estimation, and perioperative planning in urological practice.

#### 4.2.1 Methodological description and statistical analysis

We will harmonise variables by reconciling definitions of exposures, outcomes, and covariates across data sets using a standardised data dictionary. When necessary, we recoded or imputed variables to ensure consistency.

The primary outcomes (eg, major postoperative complications within 30 d and 90 d, long-term functional outcomes, survival) and secondary outcomes (eg, length of stay, readmission, minor complications) included and candidate predictors (eg, demographic factors, comorbidities, procedural variables, and preoperative laboratory parameters) will be described.

We will use a one-stage IPD meta-analysis framework that fits generalised linear mixed-effects models with study or centre as a random effect to account for clustering. This approach allows simultaneous estimation of overall effects and exploration of patient-level interactions. Missing data will be handled via multiple imputation under the assumption of missing at random. We will assess heterogeneity by estimating variance components across studies and tested for effect modification by key covariates. Sensitivity analyses will include two-stage approaches and complete-case analyses to evaluate the robustness of the findings.

### 4.3 Machine learning

Machine learning (ML) algorithms will be used to identify generalisable complication-intervention event patterns and enhance prediction accuracy beyond traditional statistical models. The objective is to identify nonlinear relationships and complex interactions that may be overlooked in conventional analyses. The intention is to use an ensemble approach that leverages a suite of ML algorithms. This suite consists of standard techniques, including logistic regression (for binary outcomes only), support vector machines, naïve Bayes classifiers, k-nearest neighbours, discriminant analysis, random forests, and neural networks. If the sample size and data richness permit, more advanced deep learning techniques may also be investigated, including recurrent neural networks or long short-term memory models for sequential or time-dependent data, autoencoders for unsupervised dimensionality reduction and anomaly detection, and Bayesian neural networks for quantification of uncertainty. Model training will use repeated tenfold cross-validation within the development data set (80%). An external validation set (20%) will be used to test generalisability. A grid of tuning parameters will be established on the basis of initial analysis, and optimal configurations will be selected by minimising the cross-validation error. The performance of models will be assessed using a comprehensive set of metrics, including discrimination measures (ROC, AUC, precision, recall, F1 score, and balanced accuracy), calibration metrics (Brier score, calibration plots, and the Hosmer-Lemeshow test, where applicable), and interpretability tools such as variable importance metrics (eg, Gini index in random forests).

### 4.4 Development of prediction models for diverse outcome parameters

Multivariate parametric prediction models will be used to assess the simultaneous effects of independent variables on a range of outcome measures. Depending on the type of outcome, we will apply multivariate linear regression for continuous variables, multivariate logistic regression for binary outcomes, and generalised linear (mixed) models for data with a non-normal distribution or hierarchical structure. Outcome measures will include mortality and morbidity-related parameters (eg, overall complication burden at defined time points, and specific complications such as ureteroileal anastomotic stricture).

When necessary, outcome transformations (eg, Box-Cox, inverse-logit) will be used to meet model assumptions such as normality of residuals or homogeneity of variance. If sufficient data are available, we will split the data set into a training set (80%) and an independent testing/validation set (20%) to allow internal validation and prevent model overfitting. Model selection will be guided by stepwise procedures (eg, backward elimination) that minimise the AIC.

Model diagnostics may include the Shapiro-Wilk test for the normality of residuals, Bartlett’s test for the homogeneity of variance, standardised residuals to detect outliers, and the Hosmer-Lemeshow test for generalised linear (mixed) models. Model performance will be evaluated using standard techniques such as ROC curves and the AUC for classification models and R^2^ values for continuous outcome models.

We will conduct sensitivity analyses by applying different link functions, excluding outliers and influential observations, or developing comparable prediction models using modern ML methods such as support vector machines, naïve Bayes, or decision trees (Section 4.5). Results will be visually presented with confidence intervals and/or summarised in tables.

### 4.5 Prediction modelling of complications

In the first phase, prediction models for complications will be developed using data from a tertiary referral centre for cystectomy and urinary diversion (Department of Urology, Inselspital Bern) that were prospectively collected between 1999 and 2021, including 1557 patients undergoing cystectomy and urinary diversion at the Department of Urology, Inselspital Bern.

In the second phase, these models will be externally validated using data from the CAMUS collaborative cohort described above.

For the Bern cystectomy cohort, each complication (with a maximum of 54) was recorded with respect to the postoperative day (POD), type of complication (eg, pulmonary, cardiac), and type of intervention required, and was graded according to the CDC as determined by two senior clinicians. Using the CDC, we calculated the CCI score for POD 1 to POD 30 for each patient. To calculate the CCI score, each CDC grade was multiplied by a specific weight as described in the original publication. These weights were derived from patients and physicians to accurately reflect the severity of complications from a clinical perspective. Multiple complication scores were then summarised for each patient. The square root of the sum divided by two yields the CCI score. The maximum value of the CCI score was set to 100, which reflects death of the patient.

To proceed with prediction modelling, all preoperative available variables will be included. Laboratory values will be used if the proportion of missing values is ∼<25% and the assessment date is within 30 d before surgery as part of the clinical routine. In cases of multiple values, the mean will be calculated and used for further computations. For the number of patients and predictor variables, no sample size calculation will be performed. All the predictors included are assessed preoperatively and will be grouped into categories according to the clinical context (eg, baseline data, laboratory findings, cancer data). Categorical predictors will then be summarised using the count and proportion, while numerical predictors will be described using the mean and median, depending on the data distribution.

The primary model outcome will be CCI-defined (and therefore cumulative) morbidity between POD 1 and POD 30, and between POD 31 and POD 90. Owing to the nature of the data assessment, blinding of the assessors is not feasible. On the basis of clinical relevance, we have defined four CCI thresholds for our analyses: ≥20.9 = mild, ≥26.2 = moderate, ≥33.7 = moderate-severe, and ≥42.4 = severe. These thresholds reflect CCI scores for CDC grade II, IIIa, IIIb, and IVa, respectively.

Prediction models will be reported according to the guidelines for reporting clinical prediction models that use regression or ML methods (TRIPOD+AI; https://www.tripod-statement.org/). The model-building and evaluation approach used has been described in previous studies on the development of clinical prediction models. To assess the predictive performance for the primary outcome, we will train random forest models owing to their ability to deal with nonlinear relationships between dependent and independent variables. This is an important feature when predicting complication risk, as nonlinearity must be expected. For each predictor category, a model will be trained for both POD and CCI thresholds.

Missing data will be imputed using a single imputation method, assuming missing at random. For continuous variables, we will use median imputation. Categorical variables will be imputed by the mode.

For each model, the data set will be randomly split into a training set (70%) and a test set (30%), with splitting performed with respect to the outcome variable. Predictor variables will not be rescaled, standardised, or transformed and no hyperparameter tuning will be performed. All models will be calculated using the *caret* package.

ROC curves and the corresponding AUC will be calculated using predicted probabilities with the *pROC* package. Confidence intervals for the AUC values will be computed via bootstrapping. Calibration belts with associated 95% confidence intervals will be created for POD 3, 5, 10, 20, and 30 using the *givitiR* package. As recommended, the clinical utility of the trained models will be assessed using decision curve analysis for selected PODs (POD 5 and 30) with different CCI thresholds for the best-performing model domain using the *dcurves* package. Decision curve analysis involves the concept of net benefit (NB). NB weights benefit from true-positive classifications, with potential harm from false-positive classifications. Specific NB values depend on the risk aversion of clinicians and/or patients, which puts model performance in a realistic clinical context. Owing to differences in the prevalence of complications according to severity and POD, the standardised NB will be calculated by dividing the NB values by the highest attainable NB in each model to allow better and more intuitive comparison between models.

### 4.6 Software and statistical power

Throughout, we will analyse data using SPSS v20.0 statistical software (SPSS, Chicago, IL, USA) and R v4.1.0 (R Foundation for Statistical Computing, Vienna, Austria,). Owing to the exploratory and retrospective nature of the studies, no formal a priori power calculation will be conducted. However, post hoc power estimates may be provided for key findings where appropriate. Missing data will be assessed, and if >5% of data are missing for key variables, a multiple imputation method (eg, multiple imputation by chained equations) will be applied. Sensitivity analyses will be conducted to compare the results from imputed and complete-case analyses. Statistical significance will be defined as *p* < 0.05 (two-tailed), unless otherwise stated.

## 5 Discussion

The CAMUS initiative addresses long-standing gaps in surgical outcome reporting by introducing a patient-inclusive, multidisciplinary, and internationally validated classification system for urological complications. Historically, complication reporting has suffered from inconsistency and under-reporting that hinder effective benchmarking, research, and informed consent. Complication rates reported after major urological procedures such as cystectomy vary widely—for instance, 30-d and 90-d morbidity rates range from 26% to 86%, and from 30% to 100%, respectively—which underscores the urgent need for a standardised methodology.

The global representation of academic centres, private hospitals, and regional surgical units within CAMUS facilitated the development of a data set that reflects real-world diversity in patient populations, surgical complexity, and health care systems. This multicentre, multinational architecture strengthens the generalisability and clinical relevance of the CAMUS classification, and provides clarity on the true burden of surgical complications across various settings and levels of care.

Drawing on data from more than 130 000 major urological procedures contributed by 180 centres across 33 countries, CAMUS represents one of the largest and most comprehensive complication data sets assembled in the field. The breadth of this registry supports robust multivariable modelling to evaluate how baseline characteristics such as comorbidities, tumour burden, and prior interventions impact postoperative outcomes. With this scale, CAMUS provides the statistical power required to develop validated prediction models and risk-adjusted benchmarks, which thus addresses the longstanding opacity in surgical morbidity and will strengthen the evidence base for shared decision-making.

Crucially, CAMUS moves beyond physician-centred grading systems such as the CDC and CCI, which either fail to capture multiple complications per patient or reduce complex trajectories to single summary scores. CAMUS has a granular, event-level reporting format that accommodates cumulative, sequential, and multimodal complications, and thus more accurately reflects the full arc of patient morbidity and will facilitate both clinical audit and research.

A defining innovation of CAMUS is the integration of nursing and patient perspectives. Nurses contribute valuable insights into early deterioration and recovery trajectories, and their participation in the Delphi consensus improved the credibility and inclusivity of the reporting system. Equally significant is the incorporation of patient-reported outcomes and behavioural economic methods such as WTP and burden scaling, which highlight the high subjective impact of complications often considered “low-grade” in clinical terms (eg, urinary incontinence or sexual dysfunction). This alignment with patient priorities enhances the utility of the system for shared decision-making and counselling.

Another major advance is the IPRADES instrument. Developed via expert consensus and validated against CAMUS data sets, this organ-specific scoring system stratifies surgical complexity and perioperative risk. Its modular design incorporates preoperative and intraoperative factors to support multidisciplinary planning, patient selection, and trainee allocation. Later phases will expand validation across additional procedures and institutions.

Beyond expert-derived indices, ML approaches may further enhance the estimation of surgical difficulty by integrating patient factors, intraoperative metrics, and outcome data at scale. Artificial intelligence–based models can identify nonlinear relationships and dynamically refine risk estimates over time. These approaches are envisioned as complementary decision-support tools that augment, rather than replace, clinically interpretable indices such as IPRADES.

### 5.1 Strengths

The CAMUS initiative is unique in both scale and scope. Its principal strength lies in its multinational, multidisciplinary, and multistakeholder design to capture perspectives from surgeons, anaesthetists, nurses, patients, and health economists. Unlike prior frameworks, CAMUS integrates both objective clinical parameters and subjective patient-reported burdens, and thus bridges a longstanding gap between clinician-centred and patient-centred outcome measures. The combination of Delphi consensus studies, behavioural economics methods, and large-scale registry validation ensures both methodological robustness and practical feasibility.

Another strength is the prospective orientation: unlike retrospective systems that suffer from heterogeneity and under-reporting, CAMUS is built on encrypted REDCap-based infrastructure, which allows structured, real-time complication capture and cross-centre benchmarking. The initiative anticipates future integration with electronic health records (EHRs), mobile applications, and ML-driven predictive tools, ensuring adaptability to evolving healthcare technologies. Collectively, CAMUS functions not only as a classification system but also as a template for global standardisation in surgical quality assurance.

Standardised definitions and intervention-based logic make CAMUS particularly amenable to automated data extraction. Rule-based algorithms, natural language processing, and ML tools can identify complication-intervention events from structured and unstructured clinical data, with prepopulation of CAMUS fields for clinician validation. This human-in-the-loop approach has the potential to substantially reduce manual data entry while improving completeness and accuracy.

### 5.2 Impact on research

CAMUS can reshape surgical outcomes research by overcoming the lack of standardisation that has historically limited cross-study comparisons. By generating the world’s largest harmonised data set of urological complications, CAMUS will allow predictive modelling, big-data analytics, and ML to explore trajectories of multimodal or recurrent complications rather than collapsing them into a single grade.

Because CAMUS incorporates nursing and patient perspectives, it can also facilitate mixed-methods studies examining how bedside recognition and subjective burden correlate with objective morbidity. Its structured methodology and dynamic data dictionary make it reproducible and scalable, which represents a prototype for complication reporting across other surgical specialties.

### 5.3 Impact on clinical practice

In clinical practice, CAMUS is poised to improve patient safety, perioperative planning, and transparency in surgical audits. The patient-centred burden index redefines what constitutes a “major” complication, which will ensure that outcomes traditionally regarded as “minor” (eg, incontinence, sexual dysfunction) are recognised for their QoL impact. This will directly strengthen informed consent and support shared decision-making.

The CAMUS IPRADES risk and difficulty score will allow structured case stratification, trainee allocation, and targeted perioperative resource planning such as ICU triage. Institutions will benefit from CAMUS-enabled dashboards that can enhance transparency in morbidity and mortality reviews, support benchmarking, and align with value-based health care frameworks. At a system level, CAMUS will provide robust, standardised metrics that can inform policy-making, reimbursement models, and surgical quality indicators.

The REDCap-based e-database, which is mapped to a dynamic ICD-10–linked dictionary, allows multilayered complication recording, PROM integration, and automated reporting. Pilot evaluations have shown high user satisfaction, and future developments will include mobile entry, EHR integration, and predictive analytics.

### 5.4 Limitations

Despite its strengths, CAMUS is not without limitations. Retrospective data collection carries risks of selection and documentation bias, and institutional variability in reporting thresholds persists. While prospective validation and structured training will be applied to mitigate these issues, full integration will require sustained cultural and operational change across institutions.

The use of behavioural economics tools in clinical contexts also introduces challenges, including cross-cultural variability in how patients interpret economic trade-offs and potential response bias. These differences highlight the importance of ongoing calibration and localisation of PROMs to ensure sensitivity, cultural appropriateness, and interpretability across diverse health care settings.

Finally, logistic and regulatory barriers to global data-sharing remain. Variations in governance frameworks, ethics approval processes, and digital infrastructure may delay widespread adoption. Nevertheless, the phased design of CAMUS—spanning retrospective analysis, Delphi consensus, e-database development, and prospective validation—ensures that these limitations are systematically addressed.

CAMUS establishes a foundation for global standardisation in complication reporting, with potential for cross-specialty expansion. Its tools support surgical planning, quality assurance, education, and longitudinal outcome monitoring. By shifting the paradigm from single-event reporting to patient-centred, multistakeholder classification, CAMUS offers a more transparent, inclusive, and clinically useful model of surgical quality. Future directions include broader procedure coverage (endoscopic and ambulatory urology), ML-enhanced risk prediction, and embedding of CAMUS within surgical training and international guidelines to ensure that its benefits extend to both clinicians and patients.

## 6 Conclusions

The CAMUS initiative has established a global, multidimensional framework for surgical complication reporting that overcomes the limitations of existing classification systems. By integrating surgeon, nurse, and patient perspectives with rigorous methodological design, CAMUS advances the accuracy, transparency, and clinical utility of postoperative morbidity documentation. Its ability to capture cumulative, sequential, and subjective complication data allows more meaningful reflection of surgical quality and patient-centred outcomes. By combining clinical, nursing, and patient-reported data within a single structured framework, CAMUS aims to standardise classification across diverse health care systems while advancing global surgical transparency.

With more than 130 000 procedures analysed and tools under development for risk stratification and assessment of intraoperative difficulty, CAMUS provides not only an advanced reporting structure but also an analytical foundation for predictive modelling, institutional benchmarking, and policy development. It can support improvements in surgical training, informed consent, and shared decision-making. The forthcoming expansion of CAMUS into prospective data capture, real-time registry platforms, and ML applications will enhance clinical decision support, audit systems, and health-service planning.

CAMUS is poised to redefine global standards in surgical reporting by promoting harmonised terminology, improving preoperative counselling, and allowing robust comparisons across centres, systems, and procedures. It represents a transformative step in the measurement of surgical outcomes that places equal weight on the perspectives of providers and patients, and that aligns surgical care with the evolving demands of transparency, accountability, and excellence in health care.

  ***Author contributions:*** Marc A. Furrer had full access to all the data in the study and takes responsibility for the integrity of the data and the accuracy of the data analysis.

  *Study concept and design:* Furrer.

*Acquisition of the data:* Soliman, Furrer.

*Analysis and interpretation of the data:* Soliman, Furrer.

*Drafting of the manuscript:* Soliman, Furrer.

*Critical revision of the manuscript of important intellectual content:* All authors.

*Statistical analysis:* Soliman.

*Obtaining funding:* Furrer, Wuethrich.

*Administrative, technical or material support:* Corcoran, Lawrentschuk, Wuethrich.

*Supervision:* Furrer.

*Other:* None.

  ***Financial disclosures*:** Marc A. Furrer certifies that all conflicts of interest, including specific financial interests and relationships and affiliations relevant to the subject matter or materials discussed in the manuscript (eg, employment/affiliation, grants or funding, consultancies, honoraria, stock ownership or options, expert testimony, royalties, or patents filed, received, or pending), are the following: None.

  ***Funding/Support and role of the sponsor*:** None.
